# Incidence of impaired kidney function among people with HIV: a systematic review and meta-analysis

**DOI:** 10.1186/s12882-022-02721-x

**Published:** 2022-03-17

**Authors:** Ruizi Shi, Xiaoxiao Chen, Haijiang Lin, Yingying Ding, Na He

**Affiliations:** 1 School of Public Health, and the Key Laboratory of Public Health Safety of Ministry of Education, Fudan University, P.O.Box 289, 138 Yi Xue Yuan Road, Shanghai, 200032 China; 2 Yiwu Research Institute of Fudan University, Shanghai, China; 3 Taizhou City Center for Disease Control and Prevention, Taizhou, Zhejiang Province China

**Keywords:** HIV, Impaired kidney function, Incidence, Prospective study, Systematic review

## Abstract

**Background:**

In the era of combination antiretroviral therapy (ART), the incidence, manifestations and severity of kidney diseases have dramatically changed in people living with HIV (PLWH). Little is known about the incidence of impaired kidney function (IKF) measured by serum creatine-based estimated glomerular filtration rate (eGFR) in PLWH.

**Methods:**

In this systematic review and meta-analysis, we searched PubMed, Ovid, Medline, Embase and Web of Science for studies published before May 7th, 2021, with estimates of incidence of IKF among PLWH. We independently reviewed each study for quality by using the Newcastle-Ottawa scale. The incidence and 95% confidence intervals (CIs) were calculated using random-effects model.

**Results:**

Sixty out of 3797 identifiable studies were eligible for the meta-analysis. A total of 19 definitions of IKF were described and categorized into three types: the threshold of eGFR, an absolute or percent decrease in eGFR, and certain eGFR threshold combined with decrement in eGFR. The eGFR< 60 ml/min/1.73m^2^ was the most widely used definition or criterion for IKF, by which the pooled incidence rate of IKF was 12.50 (95%CI: 9.00–17.36) per 1000 person years (PYs). The second most-studied outcome was a > 25% decrease in eGFR, followed by eGFR< 90 ml/min/1.73m^2^, eGFR< 30 ml/min/1.73m^2^ and a combination of eGFR threshold plus decreased eGFR. The reported incidence rates of IKF differ widely by different definitions of IKF. The highest pooled incidence was observed for those with > 25% decrease in eGFR, while the lowest was observed in those with eGFR < 30 ml/min/1.73m^2^. Substantial heterogeneity was identified across most estimates.

**Conclusion:**

Our study provides a comprehensive summary of eGFR-based definitions and incidence rates of IKF in PLWH, not only promoting our understanding of IKF, but also underscoring needs for a concerted action to unify definitions and outcomes of IKF and their applications in AIDS care.

**Supplementary Information:**

The online version contains supplementary material available at 10.1186/s12882-022-02721-x.

## Introduction

There are approximately **37.7** million people living with HIV (PLWH) worldwide, with 1.5 million newly reported in 2020 [1]. At the end of December 2020, 27.5 million PLWH were accessing antiretroviral therapy (ART) globally [1]. ART has led to considerable improvement in the life expectancy of PLWH [1–3]. Accordingly, impaired kidney function (IKF) arises as a consequence of aging, therapy of HIV infection and its complications, which is not only associated with significant morbidity and mortality in PLWH, but also their quality of life [4–6].

IKF is a generic term of various kidney dysfunctions or diseases ranging from asymptomatic changes in kidney function to severe end-stage kidney diseases (ESKD). The most typical type of the kidney disease by HIV infection before the availability of ART is HIV-associated nephropathy (HIVAN), which includes collapsing glomerulopathy, HIV-immune-complex kidney disease and thrombotic microangiopathy proved by kidney biopsy [7–9]. However, the wide use of ART over the past two decades has converted HIV infection to a chronic illness, with associated changes in the incidence, type and severity of kidney diseases in PLWH [10, 11].

IKF is characterized by a low estimated glomerular filtration rate (eGFR) or elevated urinary albumin-to-creatinine ratio (ACR) without treatment of renal replacement therapy [12, 13]. In both research and clinical practice, eGFR is largely used as an alternative for GFR that is widely accepted as the best overall indicator of kidney function [14]. Serum creatinine concentration and/or serum cystatin C are routinely used to estimate the GFR for assessment of kidney function in PLWH [7]. Although cystatin C has been proposed to be a better indicator than creatinine for IKF, previous studies support the use of the creatinine using Chronic Kidney Disease Epidemiology Collaboration (CKD-EPI) equation for routine clinical care among PLWH [15].

There is an increasing number of studies reporting the incidence of IKF measured by serum creatine-based eGFR (eGFRcreat) in PWLH across the world. However, the data have not been appropriately synthesized to provide an overview of IKF burden based on eGFRcreat in PLWH. Only one review summarized prevalence of chronic kidney disease (CKD) among PLWH [16]. To fill this gap, we synthesized available data to investigate the eGFRcreat-based incidence of IKF by different working definitions in PLWH.

## Methods

### Search strategy

In this study, a systematic literature search was performed in PubMed, Ovid Medline, EMBASE, and the Web of Science up to May 7th, 2021 to identify observational studies that reported the incidence of IKF in PLWH. A combination of the keywords and of HIV infection, IKF and incidence and their synonyms were used (Supplementary 1). We also performed a manual search for cited reference lists in the original articles and reviews.

### Study selection

Two investigators independently reviewed the identified studies for further review and meta-analysis according to preset inclusion and exclusion criteria. Duplicate records were removed before title and abstract screening. The title and abstract were first screened to confirm whether an article was potentially relevant. Then, full-texts of the articles were assessed to determine if they met the eligibility criteria. Any disagreements were addressed by the third investigator discussing with these two reviewers.

Studies were included if they fulfilled all of the following criteria: 1) reporting a certain eGFR threshold and/or eGFR decline determined by serum creatinine; 2) with a prospective or retrospective cohort study design; 3) available data for calculation; 4) with a sample size of more than 200; 5) published in English. Studies using Cockcroft-Gault formula would also be excluded given that calculation would depend on a participant’s body weight and BMI, which is different from CKD-EPI and Modification of Diet in Renal Disease (MDRD) equations.

### Quality assessment and data extraction

Two investigators independently performed quality assessment and data extraction, with discrepancy resolved by a third reviewer. Study quality was evaluated using the Newcastle-Ottawa Scale (NOS), which included three sections that covered different methodological perspectives. Scores on the NOS ranged from zero to nine points, where the higher scores indicated the better quality (Table S2). A study with a score of ≥7 was considered have high quality, with a score of 5 to 6 was considered to have moderate quality.

The following data for each study were extracted from full-text articles: first author, publication year, country or region of study, study period, male proportion, age, race composition, ART status at baseline, definition of outcome, eGFR calculation equation, frequency of eGFR measurement, sample size, duration of follow-up and numbers of incident cases.

### Statistical analysis

All definitions of IKF were summarized. The incidence of IKF was calculated by data extracted from included studies. The cumulative incidence was calculated for all studies per available incident cases and sample size. The incidence rate was only calculated for studies with available person-time. A random-effect model employing Hartung-Knapp method to adjust test statistics and confidence intervals (CIs) with Sidik-Jonkman estimator used for the between-study variance (HKSJ method) was performed to pool incidence rates, as it was proved that the HKSJ method performs better than Der Simonian-Laird (DL) method, especially when heterogeneity was present [17]. The overall incidence rates were only pooled for five most-studied definitions of IKF, including eGFR< 90 ml/min/1.73m^2^ (*n* = 3 studies), eGFR< 60 ml/min/1.73m^2^ or CKD (*n* = 28 studies), eGFR< 30 ml/min/1.73m^2^ (*n* = 6 studies), > 25% decrease in eGFR (*n* = 4 studies) and combined eGFR (short for the combination of a confirmed eGFR < 60 mL/min/1.73m^2^ with > 25% decrease in eGFR) (*n* = 4 studies), respectively. Forest plots were used to describe pooled incidence rates. We also pooled incidence rates of different stages of CKD by including all studies that defined IKF based on the cut-off values of eGFR< 60, < 50, < 45, < 30 and < 15 ml/min/1.73m^2^, respectively, in sensitive analysis.

χ2 -based Cochran’s Q test and I^2^ statistic were applied to evaluate the statistical heterogeneity across eligible studies. Cut-off values of I^2^ statistic of 25, 50, and 75% were used to define low, moderate, and high heterogeneity, respectively. When substantial heterogeneity was detected, subgroup and univariate meta-regression analysis were performed to examine sources of heterogeneity stratified by variables including WHO regions, income level, race composition, median or average age, male proportion, ART status, sample size and calculation equation. Bonferroni-adjusted *P* values were applied to univariate meta-regression for comparison of effect estimates between subgroup. Subgroup analysis, meta-regression analysis, summary of risk factors, assessment of publication bias and sensitive analysis were only conducted for pooling studies in which eGFR< 60 ml/min/1.73m^2^ was used to define IKF, as we required a minimum of ten independent studies to justify the analysis. The nonparametric “trim-and-fill” method was undertaken to assess publication bias. Publication bias was also evaluated by Begg’s (nonparametric rank correlation test) and Egger’s tests (regression-based test). *P* < 0.05 and asymmetric funnel plot indicated that there existed significant publication bias. Moreover, sensitivity analysis was performed to evaluate the influence of a single study on the overall pooled estimate by deleting one study at each step. *P* < 0.05 was considered as statistical significance. All the statistical analysis were performed using Stata 17.0 (Stata Corporation, College Station, TX, USA).

## Results

### Study selection and quality assessment

**Fig. 1:**
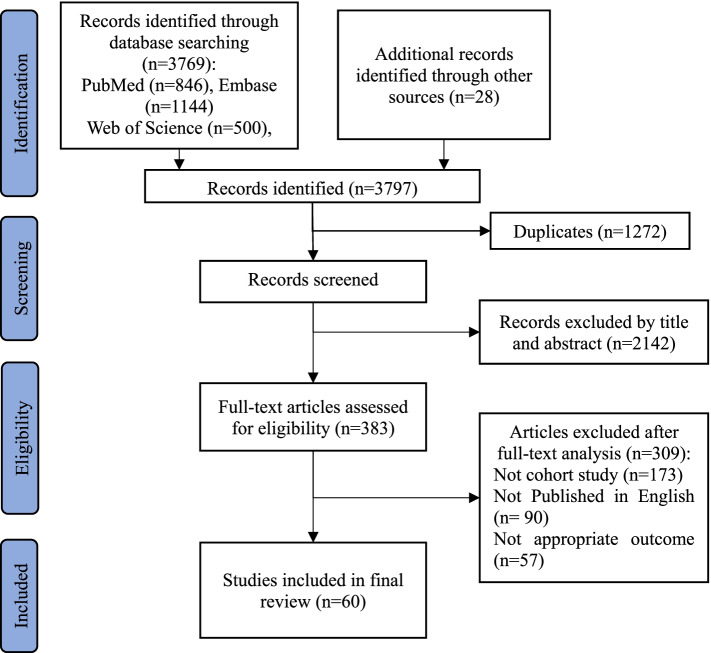
Flow diagram of study selection

A total of 3769 articles were identified through systematically searching four databases, and 28 were obtained from other sources. Of 3797 articles, 1272 articles were removed after screening for duplication. After removing 2157 unrelated articles, the 383 full-text articles were assessed for eligibility. Eventually, a total of 60 studies were processed for final review and meta-analysis. The flow chart of study selection was described in Fig. 1.

In this work, the NOS scores of most included studies ranged from six to nine points, suggesting that the quality of included studies was moderate to high (Table S2).

### Characteristics of included studies

The main characteristics of included studies are summarized in Table 1. There were 60 studies with 358,221 participants. The 60 studies were conducted in World health organization (WHO) regions as the following: African region (*n* = 7), American region (*n* = 17), European region (*n* = 15), South-east Asia region (*n* = 2), Western pacific region (*n* = 14) and multi-region studies (*n* = 5).

**Table 1:** Characteristics of the 60 studies included in the systematic review and meta-analysis by definition of outcome

Author	Year	Country	Study period	Baseline eGFR, ml/min/1.73m^**2**^	Male (%)	Sample size	Cumulative incidence (%)	No. of incident cases	eGFR equation^**‡**^
**eGFR < 90 mL/min/1.73m**^**2**^**(*n*** **= 6)**									
Lucas G. M [18].	2010	Uganda	1994–2003	no statement	35.4	1202	7	84	MDRD
Monteagudo-Chu M. O [19].	2012	USA	Dec.1998-Dec.2008	eGFR≥90	99.6	230	43	99	MDRD
Laprise C [20].	2013	Canada	Jan.2002-Mar.2012	eGFR≥90	96.2	1043	26	271	CKD-EPI
Mapesi H [21].	2018	Tanzania	Jan.2013-Jun.2016	eGFR≥90	33.3	921	12.7	117	CKD-EPI
Ding Y [22].	2019	China	Jan.2004/Dec.2014–Dec.2016	eGFR≥90	58.8	5357	16.3	872	MDRD
Liu F [23].	2020	China	Jan.2010/ Dec.2015-Jan.2017	eGFR≥90	95.4	823	21.6	178	CKD-EPI
**Decrease in eGFR > 3 mL/min/1.73m**^**2**^**(*n*** **= 2)**									
Scherzer R [24].	2012	USA	1997–2007	All range	97.7	10,841	28.4	3078	MDRD
Zachor H [25].	2016	South Africa	Sep.2010-May.2013	eGFR≥60	65.5	650	55.5	361	CKD-EPI
**CKD or eGFR < 60 mL/min/1.73m**^**2**^**(*n*** **= 40)**									
Lucas G. M [26].	2008	USA	1990-Feb.2003	eGFR > 60	68	4259	4.9	210	MDRD
Campbell L. J [27].	2009	UK	Jan.1998-Dec2006	eGFR≥60	–	1048	3.4	36	MDRD
Lucas G. M [18].	2010	Uganda	1994–2003	eGFR≥60	35.4	1202	1	12	MDRD
Flandre P [28].	2011	France	1993–2006	no statement	70.3	7378	4.7	349	MDRD
Rasch M. G [29].	2012	Denmark	Jan.1995-Jan.2009	eGFR≥60	85.2	2044	8	164	MDRD
Rockwood N [30].	2012	UK	Jun.2006-Feb.2010	eGFR> 60	87	2115	18.3	386	MDRD
Kalayjian R. C [31].	2012	USA	Apr.1996-Jul.2009	eGFR≥60	81.3	3329	3.2	106	MDRD
Scherzer R [24].	2012	USA	1997–2007	eGFR≥60	97.7	10,161	5.2	533	MDRD
Ganesan A [32]	2013	USA	HIV diagnosis-2010	eGFR≥60	92.4	3360	3.5	116	CKD-EPI
Morlat P [33].	2013	France	Jan.2004-Dec.2012	eGFR≥60	74.4	4350	4.8	209	MDRD
Lucas G. M [34].	2013	USA and Canada	1996-	eGFR > 60	85.6	59,236	11.6	6878	CKD-EPI
Scherzer R [35].	2014	USA	1997-Jan.2011	eGFR> 60	100	21,590	9.5	2059	CKD-EPI
Pujari S. N [36].	2014	UK	Jan.2007-Dec.2009	eGFR> 90	76.5	574	1.4	8	MDRD
Pujari S. N [36].	2014	India	Jan.2007-Dec.2009	eGFR> 90	75.8	708	1.6	11	MDRD
Nishijima T [37].	2014	Japan	Jan.2004/Dec.2011-Dec.2013	eGFR≥60	97	792	5.1	40	MDRD
Quesada P. R [38].	2015	Spain	Jan.2010-Dec.2012	eGFR> 60	70.1	451	14.4	65	MDRD
Estrella M. M [39].	2015	USA	Mar.2003-Mar.2012	eGFR≥60	100	333	9.9	33	CKD-EPI
Lapadula G [40].	2016	Italy	2002-Nov.2014	eGFR> 90	69	6984	2.7	191	CKD-EPI
Nishijima T [41].	2016	Japan	Jan.2004/Apr.2013-Apr.2014	eGFR> 60	96	655	0.3	2	CKD-EPI
Zachor H [25].	2016	South Africa	Sep.2010-May.2013	eGFR≥60	65.5	650	2.3	15	CKD-EPI
Hara M [42].	2017	Japan	2008–2014	eGFR≥60	90.5	544	13.2	72	MDRD
De Waal R [43].	2017	South Africa	Jul.2002-Jul.2013	no statement	37.5	15,156	7.2	1085	MDRD
Rossi C [44].	2017	Canada	Jan.2000-Dec.2012	eGFR≥60	85	2595	5.8	150	CKD-EPI
Suzuki S [45].	2017	Japan	Jan.2004-Dec.2013	eGFR≥60	94	1383	10.8	150	MDRD
Wong C [46].	2017	USA and Canada	Jan.2000-Dec.2013	eGFR> 90	83.3	52,411	3.4	1785	CKD-EPI
Bouatou Y [47].	2018	Switzerland	Jan.2002-Aug.2016	eGFR≥60	73.7	5384	4.7	252	CKD-EPI
Cheung J [48].	2018	Australia and New Zealand	Apr.2008-Mar.2016	eGFR> 60	90.7	1924	4.2	81	CKD-EPI
Jones R [49].	2018	UK	Jan.1996-Dec.2014	eGFR≥60	39.9	7764	3	231	CKD-EPI
Joshi K [50].	2018	Asian countries†	2003-Sep.2016	eGFR> 60	70.5	2547	1.5	37	CKD-EPI
Woolnough E.L. [51]	2018	D:A:D study	Dec.2009-Nov.2016	eGFR≥60	91	748	5.0	37	CKD-EPI
Pongpirul W [52].	2018	Thailand	Dec.2007-Jun.2015	eGFR≥60	58.5	5430	4.2	229	CKD-EPI
Ojeh B. V [53].	2018	Nigeria	Jan.2008-Dec.2011	eGFR≥60	32.9	5273	3.7	195	MDRD
Mapesi H [21].	2018	Tanzania	Jan.2013-Jun.2016	eGFR≥90	33.3	921	4.3	40	CKD-EPI
Matłosz B [54].	2019	Poland	1994–2015	All range	68.9	267	19.5	52	MDRD
Bock P [55].	2019	South Africa and Zambia	Jan.2014-Oct.2016	eGFR≥60	32.1	1634	1.7	27	MDRD
Kabore N. F [56].	2019	Burkina Faso	Jan.2007-Dec.2016	eGFR≥60	28	3124	0.9	27	MDRD
Domingo P [57].	2019	Spain	2010–2014	eGFR≥60	73.7	8512	2.1	183	CKD-EPI
Eaton E.F [58].	2019	USA	Jan.2007-Dec. 2014	eGFR≥60	84.0	4387	3.1	135	CKD-EPI
Mills A.M. [59]	2020	USA	Jan. 2002-Dec. 2016	eGFR > 60	84.1 84.1	22,748 22,727	5.2 5.2	1183 1182	MDRD CKD-EPI
Han W.M [60].	2020	Asian countries	Jan 2003-Mar. 2019 and Jan.2003- Sep.2017	eGFR> 60	68.3	6092	6.4	391	CKD-EPI
**eGFR < 45 mL/min/1.73m**^**2**^**(*n*** **= 3)**									
Kalayjian R. C [31].	2012	USA	Apr.1996-Jul.2009	eGFR≥60	81.3	3329	1	34	MDRD
Medapalli R. K [61].	2012	USA	No statement	eGFR≥45	97.1	12,422	9.1	1136	CKD-EPI
Suzuki S [45].	2017	Japan	Jan.2004-Dec.2013	eGFR≥60	94	1383	0.8	11	MDRD
**eGFR < 30 mL/min/1.73m**^**2**^**(*n*** **= 6)**									
Ibrahim F [13].	2012	UK	Jan.1996-Dec.2008	eGFR≥30	78.6	20,045	0.3	56	CKD-EPI
Kalayjian R. C [31].	2012	USA	Apr.1996-Jul.2009	eGFR≥60	81.3	3329	0.5	16	MDRD
Quesada P. R [38].	2015	Spain	Jan.2010-Dec.2012	eGFR≥60	70.1	451	1.1	5	MDRD
De Waal R [43].	2017	South Africa	Jul.2002-Jul.2013	no statement	37.5	15,156	1.9	292	MDRD
Suzuki S [62].	2017	Japan	Jan.2004-Dec.2013	eGFR≥60	94	1383	0.1	1	MDRD
Mapesi H [21].	2018	Tanzania	Jan.2013-Jun.2016	eGFR≥90	33.3	921	2	18	CKD-EPI
**>25% decrease in eGFR (** ***n*** **= 7)**									
Chaisiri K [63].	2010	Thailand	Jan.2007-Oct.2009	eGFR> 50	56.8	405	19.3	78	MDRD
Nishijima T [64].	2011	Japan	Jan.2002-Mar.2009	eGFR> 60	95.2	495	19.6	97	MDRD
Nishijima T [65].	2012	Japan	Jan.2004-Mar.2009	eGFR> 60	97.8	503	16.9	85	MDRD
Nishijima T [37].	2014	Japan	Jan.2004/Dec.2011-Dec.2013	eGFR≥60	97	792	34	269	MDRD
Koh H.M [66].	2016	Malaysia	Mar. 2011-Jun. 2011	all range	75.5	440	15.2	67	MDRD
Nishijima T [41].	2016	Japan	Jan.2004/Apr.2013-Apr.2014	eGFR> 60	96	655	3.1	20	CKD-EPI
Lee J. E [67].	2019	South Korea	Oct.2006-Dec.2014	eGFR≥60	88	210	12.9	27	CKD-EPI
**Confirmed eGFR < 60 among persons with baseline eGFR ≥ 60 or 25% decline in eGFR for persons with baseline eGFR < 60 (** ***n*** **= 4)**									
Low J. Z [68].	2018	Malaysia	Jan.2009-Jul.2014	eGFR≥60	89.5	314	9.6	30	MDRD
Ding Y [22].	2019	China	Jan.2004/Dec.2014–Dec.2016	All range	58.8	5357	2.4	130	MDRD
Mocroft A [69].	2020	EuroSIDA Study	Jan.2004–2018(median)	All range	74	14,754	7.7	1130	CKD-EPI
Sutton S. S [70].	2020	USA	Jan.2006-Dec.2018	All range	97	5811	15.3	889	CKD-EPI
**Decrease in eGFR > 10 mL/min/1.73m**^**2**^**(*n*** **= 3)**									
Nishijima T [37].	2014	Japan	Jan.2004/Dec.2011-Dec.2013	eGFR≥60	97	792	77.4	613	MDRD
Nishijima T [41].	2016	Japan	Jan.2004/Apr.2013-Apr.2014	eGFR> 60	96	655	80.6	528	CKD-EPI
Tan Q [71].	2019	China	Jul.2014-Apr.2015	eGFR≥60	97	258	24.8	64	CKD-EPI
**eGFR < 15 mL/min/1.73m**^**2**^**(*n*** **= 2)**									
Lucas G. M [34].	2013	USA and Canada	1996-	eGFR> 15	85.6	61,646	1.8	1098	CKD-EPI
Jones R [49].	2018	UK	Jan.1996-Dec.2014	no statement	39.9	7764	0.8	65	CKD-EPI
**Decrease in eGFR > 50% (** ***n*** **= 2)**									
Alves T. P [72].	2010	USA	Jan.1998-Dec.2005	All range	78.8	2468	2.6	63	CKD-EPI
Horberg M [73].	2010	USA	Jan.2002-Dec.2005	No statement	85.3	1674	3.9	66	MDRD
**Decrease in eGFR > 25% to < 60 mL/min/1.73 m**^**2**^**(*n*** **= 2)**									
Ando M [74].	2011	Japan	Jan.2008/Mar.2008-Jan.2009/Mar.2009	eGFR> 60	90.5	623	2.9	18	MDRD
Lucas G. M [34].	2013	USA and Canada	1996-	eGFR> 30	85.6	61,367	6.4	3945	CKD-EPI
Other definitions of impaired kidney function*									
Tordato F [75].	2011	Italy	Jan.2000–2009	All range	70	644	14.9	96	MDRD
Nishijima T [41].	2016	Japan	Jan.2004/Apr.2013-Apr.2014	eGFR> 60	96	655	6.7	44	CKD-EPI
Hara M [42].	2017	Japan	2008–2014	All range	90.5	661	77	509	MDRD
Tan Q [71].	2019	China	Jul.2014-Apr.2015	eGFR≥60	97	258	1.2	3	CKD-EPI
Dietrich L. G [76].	2020	Switzerland	Two periods with only median time	eGFR≥80	81.3	3603	6.2	225	CKD-EPI
Liu F [23]	2020	China	Jan.2010/ Dec.2015-Jan.2017	eGFR≥90	4.6	823	54.8	451	CKD-EPI
Kalemeera F [77].	2021	Namibia	Aug.2010-Dec.2016	eGFR≥60	39.6	6744	5.9	400	CKD-EPI

Note: sixteen studies reported two or more definitions of IKF at same time

MDRD, modification of diet in renal disease; CKD-EPI, Chronic kidney disease Epidemiology collaboration; NR: not report; D:A:D study, The Data Collection on Adverse Events of Anti-HIV Drugs (D:A:D) study, which is conducted in Europe, USA and Australia; EuroSIDA Study, a pan-European observational study that is conducted in Europe, Israel and Argentina.

^†^Asian countries included Cambodia, China, India, Indonesia, Japan, Malaysia, Philippines, Singapore, South Korea, Thailand and Vietnam

^**‡**^MDRD included original equations published by Modification of Diet in Renal Disease Study group and adapted equation in some countries

*Other definitions of kidney function in sequence above included: (1) advanced KDOQI category, (2) decrease in eGFR> 20%, (3) eGFR < 70 mL/min/1.73 m^2^, (4) eGFR < 50 mL/min/1.73 m^2^ or absolute decrease in eGFR > 10 mL/min/1.73 m^2^, (5) absolute decrease in eGFR > 10 mL/min/1.73 m^2^, (6) decrease in eGFR≥30%, (7) eGFR < 90 mL/min/1.73 m^2^ or decrease in eGFR > 25%, (8) eGFR < 50 mL/min/1.73 m^2^

Thirty-nine (65.0%) studies had a sample size > 1000. Fifty-eight articles reported male proportion, with a median of 81.3% (range: 4.6–76.5%). Fifty-eight (96.7%) articles reported the median or mean age, among which 23 studies had a median or mean age of ≥40 year-old. Regarding ART status at baseline, 44 articles had more than 80% of ART-experienced patients. Forty-seven articles reported the proportion of tenofovir (TDF) use, with a median of 61.0% (IQR: 48.3–100.0). Thirty-nine articles reported the proportion of protease inhibitors (PIs) use, with a median of 35.9% (IQR: 17.0–52.6).

Methods for estimates of GFR included the CKD-EPI equation (*n* = 30 studies) and the MDRD (*n* = 30 studies). More than half of included studies had two or more eGFRs to confirm the occurrence of the outcome. More information about these studies such as TDF use, PIs use at baseline or during the follow-up was presented in Table S1.

### Different definitions and incidence of IKF

Of included studies, a total of 19 definitions of IKF were described and reported (Table 1, Table S1 and Table S3), which were categorized into three types: a certain threshold of eGFR, an absolute decrease in eGFR or percent decline of eGFR and the combination of certain eGFR threshold with decrement in eGFR. The reported incidence rate of IKF differs widely by different definitions. The lowest incidence rate was observed in “eGFR< 30 ml/min/1.73m^2^”, a 0.14 per 1000 person-years (PYs) reported by Suzuki S [62]. While the highest was observed in “>25% decrease in eGFR”, with incidence rate of 190.1 per 1000 PYs [64, 78]. Specifically, 16 studies reported two or more definitions of IKF at same time.

#### eGFR< 60 ml/min/1.73m^2^ and combined eGFR

**Fig. 2:**
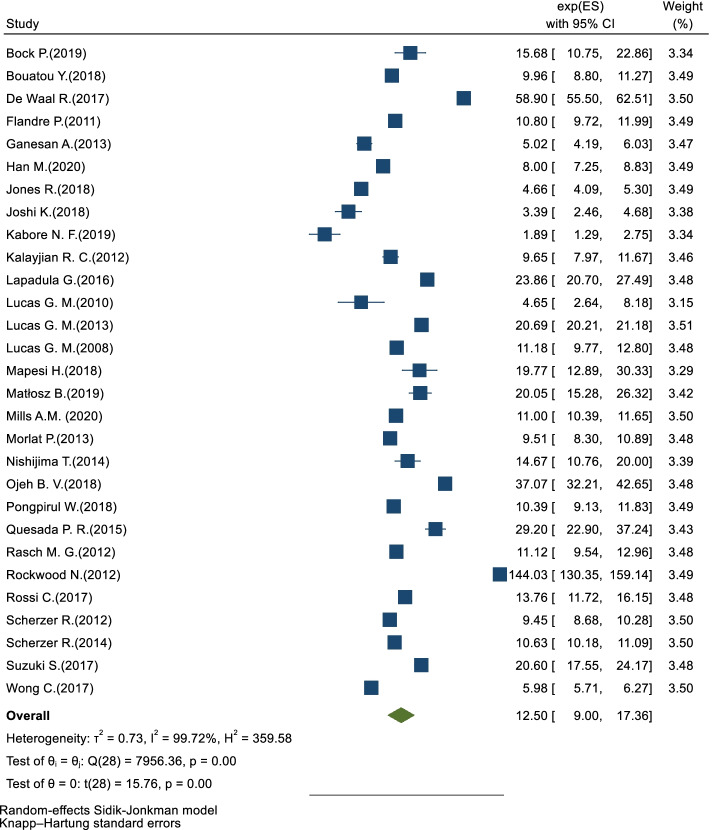
Forest plot of random-effects meta-analysis showing the pooled incidence rate of eGFR< 60 ml/min/1.73 m^2^ in PLWH

Forty studies reported the cumulative incidence of eGFR< 60 ml/min/1.73m^2^, which was the most-studied IKF in included studies. The cumulative incidence of eGFR< 60 ml/min/1.73m^2^c among PLWH ranged from 0.3 to 19.5%; the pooled incidence rate was 12.50 (95%CI: 9.00–17.36) per 1000 PYs, with an I^2^ of 99.7% **(**Fig. 2**)**. Since eGFR< 60 ml/min/1.73m^2^ was the most-studied and most-represented IKF in included studies, we summarized risk factors of it among PLWH. The risk factors reported in included studies varying from demographics (sex, age, race, height, weight, body mass index (BMI), waist-to-hip ratio (WHR), family history, and so forth), comorbidity status (viral hepatitis infection, tuberculosis (TB), hypertension (HTN), diabetes mellitus (DM), chronic obstructive pulmonary disease (COPD), dyslipidemia, lipodystrophy, and so forth) to HIV-related factors (HIV infection route, CD4 count, HIV RNA viral load, HIV diagnosis, ART regimen, and so forth) (Table S4, S5).

**Fig. 3:**
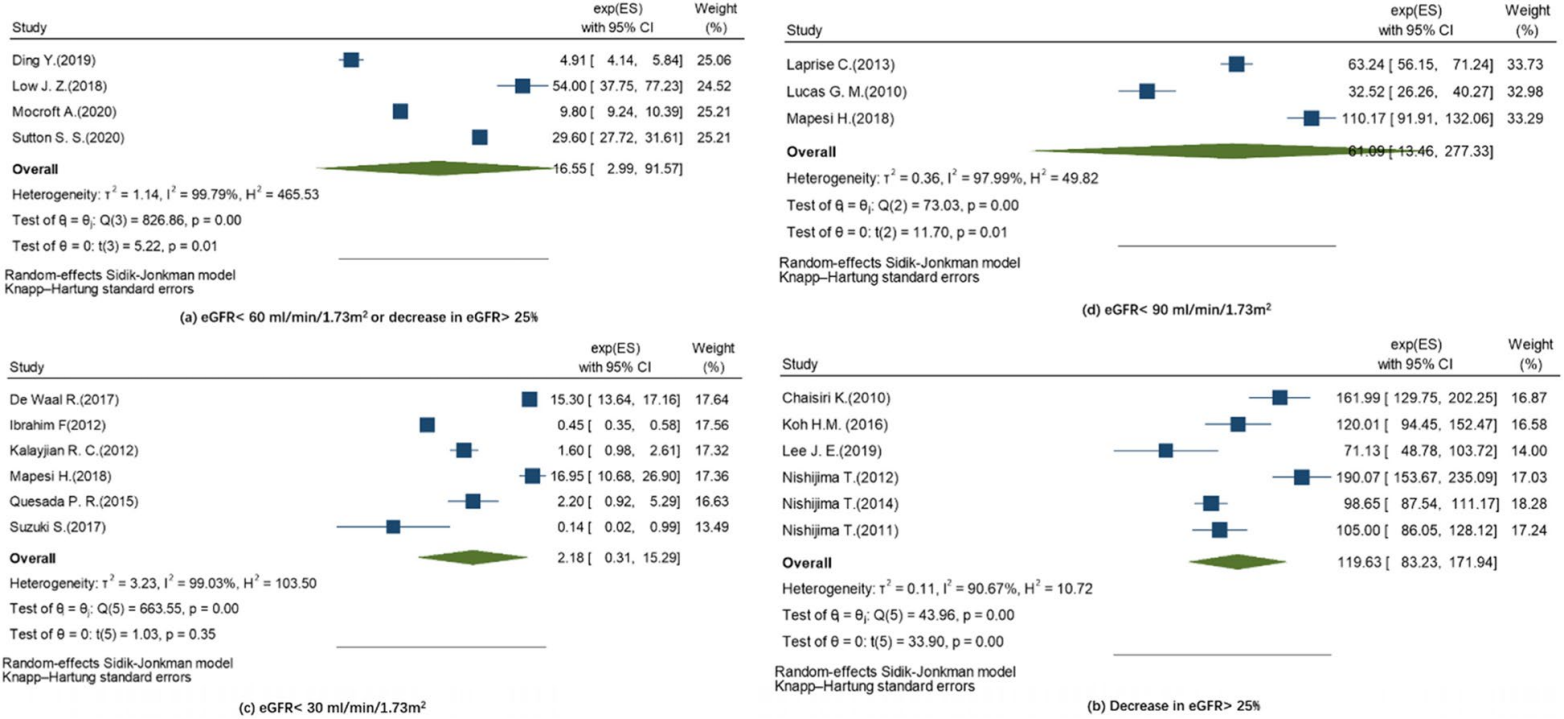
Forest plot of random-effects meta-analysis showing the pooled incidence rate of combined eGFR in PLWH

Four studies reported the result of combined eGFR with a study period of more than five years. The cumulative incidence of combined eGFR ranged from 2.4 to 15.3%. The overall incidence rate of it was 16.55 per 1000 PYs (95% CI: 2.99–91.57, I^2^ = 99.79%, *p* < 0.001)**,** which was higher than that of eGFR< 60 ml/min/1.73m^2^ but without statistical significance **(**Fig. 3**)**.

#### Decrease in eGFR > 25%

> 25% decrease in eGFR was the second most-studied IKF in included studies (*n* = 7). Seven studies reported a range of cumulative incidence from 3.1 to 34.0% (Table 1). All studies were conducted in countries in Western pacific region, four were in Japan. All studies had relatively small sample size of less than 1000 and short study period. Compared to all other definitions of pooled incidence rates, eGFR > 25% showed highest result, with 119.63 per 1000 PYs (95% CI: 83.23–171.94, I^2^ = 90.67%, *p* < 0.001).

#### eGFR< 30 ml/min/1.73m^2^

The eGFR< 30 ml/min/1.73m^2^ was the third most-studied IKF in included studies (*n* = 6). The cumulative incidence ranged from 0.1 to 2.0%, while the incidence rate ranged from 0.14 to 16.95 per 1000 PYs. Six studies were pooled for incidence rate, yielding a result of 2.18 per 1000 PYs (95%CI: 0.31–15.29, I^2^ = 99.03%, *p* < 0.001) (Fig. 3**)**.

#### eGFR< 90 ml/min/1.73m^2^

Six studies from African region (*n* = 2), American region (*n* = 2) and Western pacific region (*n* = 2) defined eGFR< 90 ml/min/1.73m^2^ as IKF. The cumulative incidence ranged from 7.0 to 43.0%. Of note, three studies had high male proportion of more than 90%, yielding three high cumulative incidence **(**Table 1**)**. The incidence rate ranged from 32.5–110.2 per 1000 PYs, and the overall incidence rate was 61.00 per 1000 PYs (95% CI: 13.46–277.33, I^2^ = 97.99%, *p* < 0.001) **(**Fig. 3**)**.

#### Other definitions of IKF

There were many other definitions of IKF in included studies, each of which contained less than three studies. However, different baseline eGFRs were applied even in studies using same definition of IKF.

Threshold of eGFR included 15, 45, 50 and 70 ml/min/1.73m^2^. More than half of these studies had sample size of more than 1000. The cumulative incidence was relatively low regardless of the study period, most of which were more than five years (Table 1). Less than half of studies reported the incidence rate but only cumulative incidence. Absolute value decreased in eGFR included 3, 10 and 20 ml/min/1.73m^2^, and most studies had sample size of less than 1000 and had high proportion of male. Two studies from Japan showed high cumulative incidence of decrease in eGFR > 10 ml/min/1.73m^2^ (77.4 and 80.6%).

Other definitions of IKF included percent decrease in eGFR and threshold of eGFR combined with percent decrease or absolute decrease in eGFR (Table 1). Most studies were from high income countries. The highest incidence rate was observed in confirmed decrease in eGFR > 20%, yielding an incidence rate of 68.0 per 1000 PYs [75].

### Subgroup analysis and meta-regression analysis

The subgroup analysis and the meta-regression analysis showed similar results, both of which identified income levels as source of heterogeneity (Table 2 and Table S6). In subgroup analysis stratified by income level, the pooled incidence rate of eGFR< 60 ml/min/1.73m^2^ in low-income countries (2.88, 95% CI: 1.24–6.71, per 1000 PYs) was lower than that in lower middle (28.08, 95% CI: 15.61–50.53, per 1000 PYs), upper middle (24.77, 95%CI: 4.53–135.37) and higher income-level groups (13.35, 95% CI: 9.63–18.50, per 1000 PYs). The incidence rate of studies using MDRD was higher than that using CKD-EPI (8.86, 95% CI: 6.20–12.64 vs. 15.41, 95% CI: 9.97–23.82, per 1000 PYs), but without statistical significance. No significant difference was detected in analysis stratified by other variables. Notably, high heterogeneity was found in nearly all subgroup analysis for eGFR< 60 ml/min/1.73m^2^ (Table 2).

**Table 2:** Subgroup analysis for incidence rate of eGFR < 60 mL/min/1.73m^2^ among PLWH

Variables	No. of study	Person-year	Case	Pooled IR (95%CI)	Test for heterogeneity		*p* for subgroup difference
					I^2^ (%)	*p*-value	
Total	29	1,346,996.15	17,133	12.50(9.13–17.11)	99.72	< 0.001	
WHO region							
African	5	41,645	1340	13.21(3.73–46.84)	99.55	< 0.001	0.025
American	9	1,052,357.45	13,020	10.05(7.65–13.2)	99.46	< 0.001	
European	9	159,452.8	1899	16.79(8.84–31.88)	99.49	< 0.001	
South-East Asia	1	61,497.5	229	10.39(9.13–11.83)	.	–	
Western Pacific	2	22,035	190	17.9(13.07–24.51)	70.15	0.056	
Mixed	3	10,008.4	455	7.5(3.2–17.57)	96.89	< 0.001	
Income level							
Low	2	16,901	39	2.88(1.24–6.71)	83.9	0.009	< 0.001
Lower middle	2	6322.9	216	28.08(15.61–50.53)	85.52	0.006	
Upper middle	2	40,456.1	1314	24.77(4.53–135.37)	99.82	< 0.001	
High	20	1,221,818.65	15,109	13.35(9.63–18.5)	99.69	< 0.001	
Mixed	3	61,497.5	455	7.5(3.2–17.57)	96.89	< 0.001	
Race^†^							
Mainly white	5	52,957.9	1058	25.67(10.03–65.72)	99.56	< 0.001	0.397
Mainly black	8	111,744	1808	11.62(5.22–25.88)	99.51	< 0.001	
Mainly Asian	5	91,818.9	847	9.74(5.38–17.65)	98.47	< 0.001	
Mixed	8	1,033,579.45	12,810	9.91(7.28–13.48)	99.57	< 0.001	
Unknow	3	56,895.9	610	12.54(8.09–19.41)	95.93	< 0.001	
Median or average age (yrs)							
< 40	17	231,407.8	2954	10.86(7.17–16.43)	99.12	< 0.001	0.019
≥40	6	550,014	9729	25.92(12.43–54.05)	99.86	< 0.001	
Unknow	6	565,574.35	4450	8.92(7.42–10.72)	97.08	< 0.001	
Male (%)							
< 60	7	92,966	1598	11.67(4.63–29.43)	99.51	< 0.001	0.861
≥60	22	1,254,030.15	15,535	12.74(9.35–17.35)	99.67	< 0.001	
ART status							
ART-experienced< 70% at baseline	7	834,670.35	10,551	8.71(5.82–13.03)	99.63	< 0.001	< 0.001
ART-experienced≥70% at baseline	11	176,674.8	1792	11.69(8.37–16.32)	97.94	< 0.001	
ART/TDF initiation	10	312,560	4674	19.09(9.3–39.22)	99.8	< 0.001	
Unknow	1	23,091	116	5.02(4.19–6.03)		.	
Sample size							
< 1000	4	8607.8	178	20.56(15.45–27.37)	71.79	0.006	0.013
≥1000	25	1,338,388.35	16,955	11.58(8.13–16.48)	99.78	< 0.001	
eGFR calculation equation							
CKD-EPI	11	691,979.4	5462	8.86(6.2–12.64)	99.3	< 0.001	0.054
MDRD	18	655,016.75	11,671	15.41(9.97–23.82)	99.74	< 0.001	

Note: IR, incidence rate; ART, antiretroviral therapy; MDRD, modification of diet in renal disease; CKD-EPI, Chronic kidney disease Epidemiology collaboration

^†^ the proportion of any race≥60% was recognized as predominant race in one study.

### Assessment of publication bias

Funnel plots and result of “trim and fill” suggested no publication bias for reporting data on the incidence rate of eGFR< 60 ml/min/1.73m^2^ (Fig. S1). Although the reporting data might underestimate the incidence rate compared with imputed result (12.50, 95%CI: 9.13–17.11 vs. 17.77, 95% CI: 12.68–24.91, per 1000 PYs), no significant difference was detected. The results were confirmed by the formal Egger test(*p* = 0.139) and Begg’s tests (*p* = 0.195).

### Sensitivity analysis

Sensitivity analysis for eGFR< 60 ml/min/1.73m^2^ by omitted one study at a time showed the stability of effect estimates (Fig. S2). Additionally, we pooled the incidence rates of all stages of CKD, including all studies with outcome defined as eGFR< 15, < 30, < 45, < 50, or < 60 ml/min/1.73m^2^, yielding an overall incidence rate of 8.43 (95% CI: 5.68–12.51), per 1000 PYs. This result was lower than the pooled result of eGFR< 60 ml/min/1.73m^2^ but without any statistical significance.

## Discussion

To our knowledge, this is the first attempt to summarize incidence estimates for IKF determined by eGFRcreat in PLWH across the world. Findings from this study revealed that various definitions of IKF based on eGFR were applied in PLWH. IKF included threshold of eGFR, absolute or percent decrease in eGFR, and certain eGFR threshold combined with decrement in eGFR. Among these outcomes, eGFR< 60 ml/min/1.73m^2^ was the most-studied and most-represented, followed by decrease in eGFR > 25%. The overall incidence rate of eGFR< 60 ml/min/1.73m^2^ was 12.50 (95%CI: 9.00–17.36) per 1000 PYs. The highest pooled incidence rate was observed in the definition of decrease in eGFR > 25%, while the lowest was observed in the eGFR < 30 ml/min/1.73m^2^, despite with substantial heterogeneity.

The eGFR< 60 ml/min/1.73m^2^ was treated as threshold of diagnosis criteria of CKD. Findings from our study suggested that most researchers still concerned about the accepted IKF---CKD. It has been suggested that the disease burden measured by eGFR< 60 ml/min/1.73m^2^ could be underestimated by only using the eGFR in comparison with studies defining the outcome as eGFR < 60 ml/min/1.73 m^2^ combined with other indicators, such as proteinuria [79]. However, our results highlighted that PLWH borne the heavy burden of IKF even if using the eGFR < 60 ml/min/1.73 m^2^. And such burden was more considerable in the late-HAART era, suggested by Jespersen, N. A., et al. [80]. The cumulative incidence and pooled incidence rate of most-reported eGFR< 60 ml/min/1.73m^2^ were approximate to the corresponding results of combined eGFR, suggested the similar disease burden. The combined eGFR was close to the definition of CKD progression in KDIGO guideline, mainly explored in EuroSIDA Study. In consideration of substantial heterogeneity in pooled results, more studies from other large cohorts are necessary to confirmed the result.

The second most-studied definition of IKF was decrease in eGFR > 25%. The highest incidence rate was observed in decrease in eGFR > 25%, which suggested that the occurrence of decrease in eGFR > 25% was most frequent in PLWH. The decrease in eGFR > 25% is more an indicator of acute kidney injury (AKI) than that of CKD [81], which is usually applied to measure the TDF-associated IKF. AKI and CKD are usually inter-connected: AKI is attributable to the development and progression of CKD; CKD is known to predispose patients to AKI [82]. However, previous study found that a large proportion of PLWH used TDF developed AKI, but only a minority of TDF users progressed to CKD [83]. In this study, we did not distinguish the CKD from AKI deliberately. Our finding suggested that the application of different definition of IKF by eGFR could reflect the predisposition of IKF in some circumstances.

The eGFR < 30 ml/min/1.73m^2^ was had lowest pooled incidence rate in this study, similar to ESRD directly diagnosed by medical record, with an incidence rate of 2.56 (95% CI, 2.33–2.81) per 1000 PYs in PLWH [55]. Previous study showed that PLWH with eGFR< 60 mL/min/1.73 m^2^ had higher risk of eGFR < 30 mL/min/1.73 m^2^ in comparison with those who with higher baseline eGFR [13]. These results stressed the importance of IKF defined by eGFR < 30 ml/min/1.73m^2^. For the eGFR< 90 ml/min/1.73m^2^, it met the criteria of mildly decreased kidney function according to the Kidney disease: Improving global outcomes (KDIGO) guidelines [14]. Mildly decreased kidney function is common in ART-experienced PLWH. However, PLWH could have improved kidney function if they switch nephrotoxic ART regimen to other regimens [84]. Moreover, they also had the possibility to transit from a mild renal impairment to a normal eGFR even they were receiving TDF [85]. However, since mild impairment can also progress to advanced stages, early detection and management of mildly IKF is essential to address the rising epidemic of kidney disease.

Of note, many other definitions of IKF were employed in studies in this review. Limited by the quantity of study, we were unable to pool the incidence rate of them, but just make a summary. Some definitions of IKF were significant indicators for certain clinical kidney disease, such as decline in eGFR ≥30%. It has been reported that the decline in eGFR≥30% was strongly associated with CKD progression to end-stage renal disease (ESRD) [86]. Therefore, it was worth paying attention to absolute or percent decrement in eGFR. However, the quantity of such studies aiming to certain definitions of IKF was small, and many of them contained only one study. More studies that investigated the present definitions of IKF were needed to confirmed their clinical significance.

Notably, in this study, there was a total of 32 selected studies from the American and European region, 14 studies from Western Pacific region, and only seven studies from Africa region where the burden of HIV infection is heavier than other regions [1]. More attention should be paid to the need for more information and validated measures of IKF in countries with heavy burden of HIV infection, especially in the context of non-communicable diseases.

The major strength of our study was the comprehensive inclusion of studies for the incidence of IKF. We searched four databases to find as many articles as possible that met our included criteria. Second, to make sure the homogeneity of study population as much as possible, we only included longitudinal studies. Any study with all participants being of coinfection or comorbidity was excluded. However, we did not limit this review to any time period (pre-highly active antiretroviral therapy (HAART) era vs. post-HAART era). Third, the quality of most of selected studies was rated as high, so our meta-analysis provided good evidence for the incidence of eGFR-based IKF. Forth, we performed the random-effect meta-analysis and the sensitivity analysis, the results of which revealed the stability of pooled incidence of IKF in PLWH.

There are limitations worth noting. First, we observed significant heterogeneity of our pooled estimates, which may be inherent to the heterogeneity between studies in terms of sample size, inclusion criteria, data collection methods, race composition and measurement of eGFR and could not be completely explained by investigated study-level characteristics. Second, as some of included studies did not provide follow-up duration, incidence rate was calculated only for those with person time. Third, limited by the number of included studies, we did not pool the incidence rate for all definitions of IKF, but just made a summary of basic characteristics and cumulative incidences. Fourth, the creatinine-based eGFR could be affected by muscle mass and diet, which might result in an overestimation of eGFR.

## Conclusion

This study revealed the substantial definitions of IKF applied in PLWH, among which eGFR< 60 ml/min/1.73m^2^ was most-studied and most-represented, followed by decrease in eGFR > 25%. These definitions would promote our understanding of IKF. Our findings also highlight the need for a concerted action to provide more evidence on certain kind of kidney impairment and a more uniform diagnostic criterion that this condition entails. Our review also points out the critical need of data from every part of the world, especially from these areas with heavy burden of HIV infection, that would help to further characterize the magnitude of CKD burden in PLWH.

## Supplementary Information

**Additional file 1.**

## Data Availability

All data generated or analyzed during this study are included in this published article and its supplementary information files.

## Abbreviations

ACR: Albumin-to-creatinine ratio
ART: Antiretroviral therapy
BMI: Body mass index
C-G: Cockcroft-Gault formula
CKD: Chronic kidney disease
CKD-EPI: Chronic kidney disease epidemiology collaboration
CIs: Confidence intervals
COPD: Chronic obstructive pulmonary disease
D:A:D study: The Data Collection on Adverse Events of Anti-HIV Drugs study
DL: Der Simonian-Laird
DM: Diabetes mellitus
eGFR: Estimated glomerular filtration rate
eGFRcreat: Serum creatine-based eGFR
ESKD: End-stage kidney diseases
EuroSIDA Study: A pan-European observational study
HAART: Highly active antiretroviral therapy
HIV: Human immunodeficiency virus
HIVAN: HIV-associated nephropathy
HKSJ: Hartung-Knapp- Sidik-Jonkman
HTN: Hypertension
IKF: Impaired kidney function
K/DOQI: The Kidney Disease Outcome Quality Initiative
MDRD: Modification of Diet in Renal Disease
NOS: Newcastle-Ottawa Scale
PLWH: People living with HIV
PIs: Protease inhibitors
TDF: Tenofovir
TB: Tuberculosis
WHO: World health organization
WHR: Waist-to-hip ratio

## References

[1] Global HIV & AIDS Statistics — 2021 Fact sheet. Joint United Nations Programme on HIV/AIDS [https://www.unaids.org/en/resources/fact-sheet.]

[2] Bärnighausen T (2015). The HIV treatment cascade and antiretroviral impact in different populations. Curr Opin HIV AIDS.

[3] Estimates of global, Regional, and national incidence, prevalence, and mortality of HIV, 1980-2015 (2016). The global burden of disease study 2015. Lancet HIV.

[4] Estrella MM, Fine DM (2010). Screening for chronic kidney disease in HIV-infected patients. Adv Chronic Kidney Dis.

[5] Kimmel PL, Barisoni L, Kopp JB (2003). Pathogenesis and treatment of HIV-associated renal diseases: lessons from clinical and animal studies, molecular pathologic correlations, and genetic investigations. Ann Intern Med.

[6] Collaboration GCKD (2020). Global, regional, and national burden of chronic kidney disease, 1990-2017: a systematic analysis for the global burden of disease study 2017. Lancet.

[7] Rosenberg AZ, Naicker S, Winkler CA, Kopp JB (2015). HIV-associated nephropathies: epidemiology, pathology, mechanisms and treatment. Nat Rev Nephrol.

[8] Nobakht E, Cohen SD, Rosenberg AZ, Kimmel PL (2016). HIV-associated immune complex kidney disease. Nat Rev Nephrol.

[9] Cohen SD, Kopp JB, Kimmel PL (2017). Kidney diseases associated with human immunodeficiency virus infection. N Engl J Med.

[10] Swanepoel CR, Atta MG, D'Agati VD, Estrella MM, Fogo AB, Naicker S (2018). Kidney disease in the setting of HIV infection: conclusions from a kidney disease: improving global outcomes (KDIGO) controversies conference. Kidney Int.

[11] Kudose S, Santoriello D, Bomback AS, Stokes MB, Batal I, Markowitz GS (2020). The spectrum of kidney biopsy findings in HIV-infected patients in the modern era. Kidney Int.

[12] George E, Lucas GM, Nadkarni GN, Fine DM, Moore R, Atta MG (2010). Kidney function and the risk of cardiovascular events in HIV-1-infected patients. AIDS.

[13] Ibrahim F, Hamzah L, Jones R, Nitsch D, Sabin C, Post FA (2012). Baseline kidney function as predictor of mortality and kidney disease progression in HIV-positive patients. Am J Kidney Dis.

[14] Levin A, Stevens PE, Bilous RW, Coresh J, De Francisco ALM, De Jong PE (2013). Kidney disease: improving global outcomes (KDIGO) CKD work group. KDIGO 2012 clinical practice guideline for the evaluation and management of chronic kidney disease. Kidney Int Suppl.

[15] Inker LA, Wyatt C, Creamer R, Hellinger J, Hotta M, Leppo M (2012). Performance of creatinine and cystatin C GFR estimating equations in an HIV-positive population on antiretrovirals. J Acquir Immune Defic Syndr.

[16] Ekrikpo UE, Kengne AP, Bello AK, Effa EE, Noubiap JJ, Salako BL, et al. Chronic kidney disease in the global adult HIV-infected population: a systematic review and meta-analysis. PLoS One. 2018;13(4). 10.1371/journal.pone.0195443 PMC5901989 29659605

[17] IntHout J, Ioannidis JPA, Borm GF (2014). The Hartung-Knapp-Sidik-Jonkman method for random effects meta-analysis is straightforward and considerably outperforms the standard DerSimonian-Laird method. BMC Med Res Methodol.

[18] Lucas GM, Clarke W, Kagaayi J, Atta MG, Fine DM, Laeyendecker O (2010). Decreased kidney function in a community-based cohort of HIV-infected and HIV-negative individuals in Rakai, Uganda. J Acquir Immune Defic Syndr Hum Retrovirol.

[19] Monteagudo-Chu MO, Chang MH, Fung HB, Brau N (2012). Renal toxicity of long-term therapy with tenofovir in HIV-infected patients. J Pharm Pract.

[20] Laprise C, Baril JG, Dufresne S, Trottier H (2013). Association between tenofovir exposure and reduced kidney function in a cohort of HIV-positive patients: results from 10 years of follow-up. Clin Infect Dis.

[21] Mapesi H, Kalinjuma AV, Ngerecha A, Franzeck F, Hatz C, Tanner M (2018). Prevalence and evolution of renal impairment in people living with HIV in rural Tanzania. Open Forum Infect Dis.

[22] Ding Y, Duan S, Ye R, Yao S, Cao D, Yang Y (2019). Effects of aging, baseline renal function and stage of HIV infection on post-treatment changes in renal function among HIV-infected patients: a retrospective cohort study. HIV Med.

[23] Liu F, Xu A, Zhao H, Yang Z, Chen C, Ranieri B (2020). Longitudinal progression of estimated GFR in HIV-1-infected patients with normal renal function on tenofovir-based therapy in China. Ther Clin Risk Manag.

[24] Scherzer R, Estrella M, Li Y, Choi AI, Deeks SG, Grunfeld C (2012). Association of tenofovir exposure with kidney disease risk in HIV infection. AIDS.

[25] Zachor H, Machekano R, Estrella MM, Veldkamp PJ, Zeier MD, Uthman OA (2016). Incidence of stage 3 chronic kidney disease and progression on tenofovir-based regimens. AIDS.

[26] Lucas GM, Lau B, Atta MG, Fine DM, Keruly J, Moore RD (2008). Chronic kidney disease incidence, and progression to end-stage renal disease, in HIV-infected individuals: a tale of two races. J Infect Dis.

[27] Campbell LJ, Ibrahim F, Fisher M, Holt SG, Hendry BM, Post FA (2009). Spectrum of chronic kidney disease in HIV-infected patients. HIV Med.

[28] Flandre P, Pugliese P, Cuzin L, Bagnis CI, Tack I, Cabie A (2011). Risk factors of chronic kidney disease in HIV-infected patients. Clin J Am Soc Nephrol.

[29] Rasch MG, Engsig FN, Feldt-Rasmussen B, Kirk O, Kronborg G, Pedersen C (2012). Renal function and incidence of chronic kidney disease in HIV patients: a Danish cohort study. Scand J Infect Dis.

[30] Rockwood N, Mandalia S, Sirsokosta J, Gazzard B, Nelson M (2012). A comparative analysis of risk factors associated with renal impairment and highly active antiretroviral therapy. J Antivir Antiretrovir.

[31] Kalayjian RC, Lau B, Mechekano RN, Crane HM, Rodriguez B, Salata RA (2012). Risk factors for chronic kidney disease in a large cohort of HIV-1 infected individuals initiating antiretroviral therapy in routine care. AIDS.

[32] Ganesan A, Krantz EM, Hullsiek KH, Riddle MS, Weintrob AC, Lalani T (2013). Determinants of incident chronic kidney disease and progression in a cohort of HIV-infected persons with unrestricted access to health care. HIV Med.

[33] Morlat P, Vivot A, Vandenhende MA, Dauchy FA, Asselineau J, Déti E (2013). 【危险因素-诸多】role of traditional risk factors and antiretroviral drugs in the incidence of chronic kidney disease, ANRS CO3 Aquitaine cohort, France, 2004-2012. PLoS One.

[34] Lucas GM, Jing Y, Sulkowski M, Abraham AG, Estrella MM, Atta MG (2013). Hepatitis C viremia and the risk of chronic kidney disease in HIV-infected individuals. J Infect Dis.

[35] Scherzer R, Gandhi M, Estrella MM, Tien PC, Deeks SG, Grunfeld C (2014). A chronic kidney disease risk score to determine tenofovir safety in a prospective cohort of HIV-positive male veterans. AIDS.

[36] Pujari SN, Smith C, Makane A, Youle M, Johnson M, Bele V (2014). Higher risk of renal impairment associated with tenofovir use amongst people living with HIV in India: a comparative cohort analysis between Western India and United Kingdom. BMC Infect Dis.

[37] Nishijima T, Kawasaki Y, Tanaka N, Mizushima D, Aoki T, Watanabe K (2014). Long-term exposure to tenofovir continuously decrease renal function in HIV-1-infected patients with low body weight: results from 10 years of follow-up. AIDS.

[38] Quesada PR, Esteban LL, Garcia JR, Sanchez RV, Garcia TM, Alonso-Vega GG (2015). Incidence and risk factors for tenofovir-associated renal toxicity in HIV-infected patients. Int J Clin Pharm.

[39] Estrella MM, Li M, Tin A, Abraham AG, Shlipak MG, Penugonda S (2015). The association between APOL1 risk alleles and longitudinal kidney function differs by HIV viral suppression status. Clin Infect Dis.

[40] Lapadula G, Bernasconi DP, Casari S, Maggiolo F, Cauda R, Di Pietro M (2016). Risk of chronic kidney disease among patients developing mild renal impairment during Tenofovir-containing antiretroviral treatment. PLoS ONE [Electronic Resource].

[41] Nishijima T, Kurosawa T, Tanaka N, Kawasaki Y, Kikuchi Y, Oka S (2016). Urinary beta2 microglobulin can predict tenofovir disoproxil fumarate-related renal dysfunction in HIV-1-infected patients who initiate tenofovir disoproxil fumarate-containing antiretroviral therapy. AIDS.

[42] Hara M, Yanagisawa N, Ohta A, Momoki K, Tsuchiya K, Nitta K (2017). Increased non-HDL-C level linked with a rapid rate of renal function decline in HIV-infected patients. Clin Exp Nephrol.

[43] De Waal R, Cohen K, Fox MP, Stinson K, Maartens G, Boulle A (2017). Changes in estimated glomerular filtration rate over time in south African HIV-1-infected patients receiving tenofovir: a retrospective cohort study. J Int AIDS Soc.

[44] Rossi C, Raboud J, Walmsley S, Cooper C, Antoniou T, Burchell AN (2017). Hepatitis C co-infection is associated with an increased risk of incident chronic kidney disease in HIV-infected patients initiating combination antiretroviral therapy. BMC Infect Dis.

[45] Suzuki S, Nishijima T, Kawasaki Y, Kurosawa T, Mutoh Y, Kikuchi Y (2017). Effect of Tenofovir Disoproxil fumarate on incidence of chronic kidney disease and rate of estimated glomerular filtration rate decrement in HIV-1-infected treatment-Naïve Asian patients: results from 12-year observational cohort. AIDS Patient Care STDs.

[46] Wong C, Gange SJ, Buchacz K, Moore RD, Justice AC, Horberg MA (2017). First occurrence of diabetes, chronic kidney disease, and hypertension among north American HIV-infected adults, 2000-2013. Clin Infect Dis.

[47] Bouatou Y, Ageron AG, Bernasconi E, Battegay M, Hoffmann M, Staehelin C (2018). Lipodystrophy increases the risk of CKD development in HIV-positive patients in Switzerland: the LIPOKID study. Kidney Int Rep.

[48] Cheung J, Puhr R, Petoumenos K, Cooper DA, Woolley I, Gunathilake M (2018). Chronic kidney disease in Australian human immunodeficiency virus-infected patients: analysis of the Australian HIV observational database. Nephrology.

[49] Jones R, Hamzah L, Williams D, Winston A, Burns F, Phillips AN (2018). Chronic kidney disease risk in African and Caribbean populations with HIV. J Infect Dis.

[50] Joshi K, Boettiger D, Kerr S, Nishijima T, Van Nguyen K, Ly PS (2018). Changes in renal function with long-term exposure to antiretroviral therapy in HIV-infected adults in Asia. Pharmacoepidemiol Drug Saf.

[51] Woolnough EL, Hoy JF, Cheng AC, Walker RG, Chrysostomou A, Woolley I (2018). Predictors of chronic kidney disease and utility of risk prediction scores in HIV-positive individuals. AIDS.

[52] Pongpirul W, Pongpirul K, Ananworanich J, Klinbuayaem V, Avihingsanon A, Prasithsirikul W (2018). 【危险因素-DM/高TC/age/VL】chronic kidney disease incidence and survival of Thai HIV-infected patients. AIDS.

[53] Ojeh BV, Abah IO, Ugoagwu P, Agaba PA, Agbaji OO, Gyang SS (2018). Incidence and predictors of tenofovir disoproxil fumarate-induced renal impairment in HIV infected nigerian patients. Germs.

[54] Matłosz B, Pietraszkiewicz E, Firląg-Burkacka E, Grycner E, Horban A, Kowalska JD (2019). Risk factors for kidney disease among HIV-1 positive persons in the methadone program. Clin Exp Nephrol.

[55] Althoff KN, McGinnis KA, Wyatt CM, Freiberg MS, Gilbert C, Oursler KK (2015). Comparison of risk and age at diagnosis of myocardial infarction, end-stage renal disease, and non-AIDS-defining cancer in HIV-infected versus uninfected adults. Clin Infect Dis.

[56] Kabore NF, Poda A, Zoungrana J, Da O, Ciaffi L, Semde A, et al. Chronic kidney disease and HIV in the era of antiretroviral treatment: findings from a 10-year cohort study in a west African setting. BMC Nephrol. 2019;20. 10.1186/s12882-019-1335-9 PMC6505177 31064340

[57] Domingo P, Suarez-Lozano I, Gutierrez F, Estrada V, Knobel H, Palacios R (2019). Predictive factors of renal impairment in HIV-infected patients on antiretroviral therapy: results from the VACH longitudinal cohort study. Nefrologia.

[58] Eaton EF, Tamhane A, Davy-Mendez T, Moore RD, Mathews WC, Saag MS (2019). Brief report: kidney dysfunction does not contribute significantly to antiretroviral therapy modification in treatment-naive PLWH receiving initial ART. J Acquir Immune Defic Syndr.

[59] Mills AM, Schulman KL, Fusco JS, Brunet L, Hsu R, Beyer A (2020). Validation of the data collection on adverse events of anti-HIV drugs (D:a:D) chronic kidney disease risk score in HIV-infected patients in the USA. HIV Med.

[60] Han WM, Bijker R, Chandrasekaran E, Pujari S, Ng OT, Ly PS (2020). Validation of the D: a: D chronic kidney disease risk score model among people living with HIV in the Asia-Pacific. J Acquir Immune Defic Syndr.

[61] Medapalli RK, Parikh CR, Gordon K, Brown ST, Butt AA, Gibert CL (2012). Comorbid diabetes and the risk of progressive chronic kidney disease in HIV-infected adults: data from the veterans aging cohort study. J Acquir Immune Defic Syndr.

[62] Suzuki S, Nishijima T, Kawasaki Y, Kurosawa T, Mutoh Y, Kikuchi Y (2017). Effect of tenofovir disoproxil fumarate on incidence of chronic kidney disease and rate of estimated glomerular filtration rate decrement in HIV-1-infected treatment-naive Asian patients: results from 12-year observational cohort. AIDS Patient Care STDs.

[63] Chaisiri K, Bowonwatanuwong C, Kasettratat N, Kiertiburanakul S (2010). Incidence and risk factors for tenofovir-associated renal function decline among Thai HIV-infected patients with low-body weight. Curr HIV Res.

[64] Nishijima T, Komatsu H, Gatanaga H, Aoki T, Watanabe K, Kinai E (2011). Impact of small body weight on tenofovir-associated renal dysfunction in HIV-infected patients: a retrospective cohort study of Japanese patients. PLoS One.

[65] Nishijima T, Gatanaga H, Komatsu H, Tsukada K, Shimbo T, Aoki T (2012). Renal function declines more in tenofovir- than abacavir-based antiretroviral therapy in low-body weight treatment-naïve patients with HIV infection. PLoS One.

[66] Koh HM, Suresh K (2016). Tenofovir-induced nephrotoxicity: a retrospective cohort study. Med J Malaysia.

[67] Lee JE, Lee S, Song SH, Kwak IS, Lee SH (2019). Incidence and risk factors for tenofovir-associated nephrotoxicity among human immunodeficiency virus-infected patients in Korea. Korean J Intern Med.

[68] Low JZ, Khoo SP, Nor Azmi N, Chong ML, Sulaiman H, Azwa I (2018). Is the risk of tenofovir-induced nephrotoxicity similar in treatment-naive compared to treatment-experienced patients?. J Pharm Pract Res.

[69] Mocroft A, Ryom L, Oprea C, Li Q, Rauch A, Boesecke C (2020). Influence of hepatitis C virus co-infection and hepatitis C virus treatment on risk of chronic kidney disease in HIV-positive persons. AIDS.

[70] Sutton SS, Magagnoli J, Hardin JW, Hsu LI, Beaubrun A, Majethia S (2020). Association of tenofovir disoproxil fumarate exposure with chronic kidney disease and osteoporotic fracture in US veterans with HIV. Curr Med Res Opin.

[71] Tan Q, He YH, Yang TT, Yan DM, Wang Y, Zhao X (2019). Effects of long-term exposure to tenofovir disoproxil fumarate-containing antiretroviral therapy on renal function in HIV-positive Chinese patients. J Microbiol Immunol Infect.

[72] Alves TP, Hulgan T, Wu P, Sterling TR, Stinnette SE, Rebeiro PF (2010). Race, kidney disease progression, and mortality risk in HIV-infected persons. Clin J Am Soc Nephrol.

[73] Horberg M, Tang B, Towner W, Silverberg M, Bersoff-Matcha S, Hurley L (2010). Impact of tenofovir on renal function in HIV-infected, antiretroviral-naive patients. J Acquir Immune Defic Syndr.

[74] Ando M, Yanagisawa N, Ajisawa A, Tsuchiya K, Nitta K (2011). A simple model for predicting incidence of chronic kidney disease in HIV-infected patients. Clin Exp Nephrol.

[75] Tordato F, Cozzi Lepri A, Cicconi P, De Luca A, Antinori A, Colangeli V (2011). Evaluation of glomerular filtration rate in HIV-1-infected patients before and after combined antiretroviral therapy exposure. HIV Med.

[76] Dietrich LG, Thorball CW, Ryom L, Burkhalter F, Hasse B, Thurnheer MC, et al. Rapid Progression of Kidney Dysfunction in Swiss People Living with HIV: Contribution of Polygenic Risk Score and D:A:D Clinical Risk Score. J Infect Dis. 2020;05. 10.1093/infdis/jiaa695 33151293

[77] Kalemeera F, Godman B, Stergachis A, Rennie T (2021). Tenofovir disoproxil fumarate associated nephrotoxicity: a retrospective cohort study at two referral hospitals in Namibia. Pharmacoepidemiol Drug Saf.

[78] Kamkuemah M, Kaplan R, Bekker LG, Little F, Myer L (2015). Renal impairment in HIV-infected patients initiating tenofovir-containing antiretroviral therapy regimens in a primary healthcare setting in South Africa. Tropical Med Int Health.

[79] Sonoda H, Nakamura K, Tamakoshi A (2019). Ankle-brachial index is a predictor of future incident chronic kidney disease in a general Japanese population. J Atheroscler Thromb.

[80] Jespersen NA, Axelsen F, Dollerup J, Nørgaard M, Larsen CS (2021). The burden of non-communicable diseases and mortality in people living with HIV (PLHIV) in the pre-, early- and late-HAART era. HIV Med.

[81] Bellomo R, Ronco C, Kellum JA, Mehta RL, Palevsky P (2004). Acute renal failure - definition, outcome measures, animal models, fluid therapy and information technology needs: the second international consensus conference of the acute Dialysis quality initiative (ADQI) group. Crit Care.

[82] He L, Wei Q, Liu J, Yi M, Liu Y, Liu H (2017). AKI on CKD: heightened injury, suppressed repair, and the underlying mechanisms. Kidney Int.

[83] Wikman P, Safont P, Del Palacio M, Moreno A, Moreno S, Casado JL (2013). The significance of antiretroviral-associated acute kidney injury in a cohort of ambulatory human immunodeficiency virus-infected patients. Nephrol Dial Transplant.

[84] Jose S, Nelson M, Phillips A, Chadwick D, Trevelion R, Jones R (2017). Improved kidney function in patients who switch their protease inhibitor from atazanavir or lopinavir to darunavir. AIDS.

[85] De Beaudrap P, Diallo MB, Landman R, Gueye NF, Ndiaye I, Diouf A (2010). Changes in the renal function after tenofovir-containing antiretroviral therapy initiation in a Senegalese cohort (ANRS 1215). AIDS Res Hum Retrovir.

[86] Coresh J, Turin TC, Matsushita K, Sang Y, Ballew SH, Appel LJ (2014). Decline in estimated glomerular filtration rate and subsequent risk of end-stage renal disease and mortality. JAMA.

