# Supplementary material for: Incidence of impaired kidney function among people with HIV: a systematic review and meta-analysis
Source: BMC Nephrol. 2022 Mar 17;23:107. doi: 10.1186/s12882-022-02721-x (PMC8932163; doi:10.1186/s12882-022-02721-x)
Supplement: Supplementary file 1 — Additional file 1. [file 12882_2022_2721_MOESM1_ESM.docx]

**Supplementary 1 Search strategy**

**PubMed：**

(("Epidemiology"[Title/Abstract] OR "inciden*"[Title/Abstract] OR "Cohort" [Title/Abstract]) AND (((renal insufficiency [MeSH Terms] OR Kidney Insufficienc*[Title/Abstract] OR Renal Insufficienc* [Title/Abstract] OR renal function [Title/Abstract] OR kidney function [Title/Abstract] OR renal impairment [Title/Abstract] OR kidney impairment [Title/Abstract] OR renal Diseas* [Title/Abstract] OR Kidney Diseas* [Title/Abstract] OR renal dysfunction [Title/Abstract] OR kidney dysfunction [Title/Abstract] OR Renal Failur* [Title/Abstract] OR Kidney Failur* [Title/Abstract] OR End Stage Kidney* [Title/Abstract] OR End Stage Renal* [Title/Abstract] OR ES?D [Title/Abstract]) AND (HIV[Title/Abstract] OR Human Immunodeficiency Virus [Title/Abstract] OR AIDS Virus [Title/Abstract] OR Acquired Immune Deficiency Syndrome* [Title/Abstract])) AND ("Epidemiolog*"[Mesh] OR "Inciden*"[Mesh] OR "Cohort*"[Mesh]))) AND (HIV[Title/Abstract] OR Human Immunodeficiency Virus [Title/Abstract] OR AIDS Virus [Title/Abstract] OR Acquired Immune Deficiency Syndrome* [Title/Abstract])

**Ovid Medline:**

(HIV infection.mp. or exp HIV Infections/ exp HIV/ or hiv.mp. OR AIDS.mp. or exp Acquired Immunodeficiency Syndrome/ OR HIV.ab,kf,sy,ti. OR Human immunodeficiency virus.ab,kf,sy,ti. OR AIDS.ab,kf,sy,ti. OR "Acquired Immune Deficiency Syndrome*".ab,kf,sy,ti.) AND (exp renal insufficiency, chronic/ or exp kidney failure, chronic/ OR Renal Insufficienc*.mp. OR ESRD.mp. OR end stage renal*.mp. OR renal impairment.mp. OR kidney impairment.mp. OR "kidney Insufficienc*".mp. OR Renal Insufficienc*.mp. OR kidney dysfunction.mp. OR renal dysfunction.mp. OR kidney function.mp. OR renal function.mp. OR End Stage Kidney*.mp. OR "End Stage renal*".mp.) AND (incidence.mp. or exp Incidence/ OR cohort studies.mp. or exp Cohort Studies/ OR epidemiology.ab,kf,sy,ti. OR "incidence".ab,kf,sy,ti. OR "cohort stud*".ab,kf,sy,ti.)

**Embase:**

(HIV infection.mp. or exp Human immunodeficiency virus infection/ OR hiv.mp. or exp Human immunodeficiency virus/ OR AIDS.mp. or exp acquired immune deficiency syndrome/ OR HIV.ab,kw,ti. OR Human immunodeficiency virus.ab,kw,ti. OR AIDS.ab,kw,ti. OR Acquired Immune Deficiency Syndrome Virus.ab,kw,ti.) AND (Renal Insufficienc*.mp. OR Renal impairmtnet.mp. OR kidney dysfunction.mp. OR renal dysfunction.mp. OR ESRD.mp OR "Renal Insufficienc*".ab,kw,ti. OR "kidney Insufficienc*".ab,kw,ti. OR kidney dysfunction.ab,kw,ti. OR renal dysfunction.ab,kw,ti. OR kidney impairment.ab,kw,ti. OR renal impairment.ab,kw,ti. OR "End Stage Kidney*".ab,kw,ti. OR "End Stage renal*".ab,kw,ti.) AND (incidence.ab,kw,ti. OR cohort.ab,kw,ti. OR epidemiology.ab,kw,ti. OR exp cohort analysis/ or cohort stud*.mp. or exp Cohort Studies/ OR incidence rate.mp. or exp incidence/)

**Web of Science:**

(TS=(HIV) OR TS=(AIDS) OR TS=(Human Immunodeficiency Viruses) OR TS=(Acquired Immunodeficiency Syndrome) OR TI=(HIV) OR TI=(AIDS) OR TI=(Human Immunodeficiency Viruses) OR TI=(Acquired Immunodeficiency Syndrome) OR AB=(HIV) OR AB=(AIDS) OR AB=(Acquired Immunodeficiency Syndrome) OR AB=( Human Immunodeficiency Viruses) OR TS=( antiretroviral therapy) OR TI=( antiretroviral therapy) OR AB=( antiretroviral therapy) OR TS=(HAART) OR TI=( HAART) OR AB=( HAART)) AND (TS=(renal insufficienc*) OR TI=( renal insufficienc*) OR AB=( renal insufficienc*) OR TS=(renal function) OR TI=( renal function) OR AB=( renal function) OR TS=( Kidney Insufficienc*) OR TI=( Kidney Insufficienc*) OR AB=( Kidney Insufficienc*) OR TS=(kidney function) OR TI=(kidney function) OR AB=( kidney function) OR TS=( renal Diseas*) OR TI=( renal Diseas*) OR AB=( renal Diseas*) OR TS=( Kidney Diseas*) OR TI=( Kidney Diseas*) OR AB=( Kidney Diseas*) OR TS=( renal dysfunction) OR TI=( renal dysfunction) OR AB=( renal dysfunction) OR TS=( kidney dysfunction) OR TI=( kidney dysfunction) OR AB=( kidney dysfunction) OR TS=( Renal Failur*) OR TI=( renal Failur*) OR AB=( renal Failur*) OR TS=( Kidney Failur*) OR TI=( Kidney Failur*) OR AB=( Kidney Failur*) OR TS=( End Stage Renal *) OR TI=( End Stage Renal *) OR AB=( End Stage Renal *) OR TS=( End Stage Kidney *) OR TI=( End Stage Kidney *) OR AB=( End Stage Kidney *) OR TS=( ES?D) OR TI=( ES?D) OR AB=( ES?D) OR TS=(renal impairment) OR TI=( renal impairment) OR AB=( renal impairment) OR TS=( kidney impairment) OR TI=( kidney impairment) OR AB=( kidney impairment)) AND (TS=(Epidemiolog*) OR TI=(Epidemiolog*) OR AB=(Epidemiolog*) OR TS=( Incid *) OR TI=( Incid *) OR AB=( Incid *) OR TS=( Cohort Stud *) OR TI=( Cohort Stud *) OR AB=( Cohort Stud *))

**Supplementary Table 1. Characteristics of the studies included in the systematic review and meta-analysis**

| **Author** | **Year** | **WHO Region/**  **income level** | **Outcome*^#^ (mL/min/1.73m^2^)** | **Age**  **(years)** | **Male (%)** | **Race composition** | **ART at Baseline (%)** | **TDF use (%)** | **PIs use (%)** | **Sample size** | **Follow-up (PY)** | **No. of cases** | **Incidence rate (per 1000 PYs)** |
| --- | --- | --- | --- | --- | --- | --- | --- | --- | --- | --- | --- | --- | --- |
| Alves, T. P. | 2010 | PAHO/ High | ≥50% decrease in eGFR | median: non-AA:38, AA:39 | 78.8% | black 33.2% others 66.7% | 40.4% at baseline | NR | NR | 2468 | median:2.1 (range: 0.25-7.9) years | 63 | - |
| Ando, M. | 2011 | WPRO/High | CKD (2) and >25% decrease in eGFR (2); | CKD:58 (52–64), non-CKD 43 (36–55) | 90.5% | Asian | 95.70% | 57.00% | 52.65% | 623 | 1 year | 18 | - |
| Bock P. | 2019 | mixed | CKD (1) | 31 (IQR:26–38) | 32.1% | unclear but probably black | TDF initiation | 100.0% | NR | 1634 | 1722 | 27 | 1.57 |
| Bouatou Y. | 2018 | EURO/ High | CKD within 6 months (2) | 38.8±10.7 (median:38, range:16-80) | 73.7% | white 72.6% black 17.5%, Hispano-American 4.7% Asian 5.0%, others 0.2% | 49.1% | 28.1% | 21.5% | 5384 | 25301.9 | 252 | 9.96 |
| Campbell, L. J. | 2009 | EURO/ High | CKD (2s) | mean <40 |  | white/other 61.3%, black/mixed black 38.7% | 100.0% | 80.4% | 19.6% | 1048 | NR | 14 for IDV, 22 for TFV | - |
| Chaisiri, K. | 2010 | SEARO/ Upper middle | >25% decrease in eGFR (1s) | 40 (IQR:36-45) | 56.8% | Asian | 99% at baseline and TDF initiation | 100.0% | 29.1% | 405 | 481.5 | 78 | 162.00 |
| Cheung J. | 2018 | WPRO/High | CKD (3s) | no-CKD:45(IQR:38-52), CKD:57(IQR:51-64) | 90.7% | Asian | 97.7% | 51.2% | 17.0% | 1924 | NR | 81 | - |
| De Waal, R. | 2017 | AFRO/ Upper middle | ① CKD (1s); ② eGFR <30 (1s) | 35.4 (IQR:29.9-42.0) | 37.5% | unclear but mainly black | TDF initiation | 100.0% | NR | 15156 | ①18421.1; ②19085 | ①1085,②292 | ①58.9; ②15.3 |
| Dietrich, L. G. | 2020 | EURO/ High | Annual decrease in eGFR >5 unit (3s) | outcome: 42(IQR:37-50); non:42(IQR:37-48) | 81.3% | unclear but mainly white | NR | only duration of treatment | only duration of treatment | 3603 | NR | 225 | - |
| Ding Y. | 2019 | WPRO/ Upper middle | ①CKD (2) or >25% decrease in eGFR (2); ②eGFR: 60-89 (2) | 35.5 (IQR:29.6–42.5) | 58.8% | Asian | 100.0% | NR | NR | 5357 | ①26457; ②NR | 130 | 4.9 |
| Domingo P. | 2019 | EURO/ High | CKD (NR) | 44 (IQR:39-48) | 73.7% | white 85.3%, black 3.3%, others 11.5% | 94.3% | 57.0% | 43.2% | 8512 | NR | 183 | - |
| Eaton, E.F. | 2019 | PAHO/ High | CKD (1) | age category | 84.0% | white 40%, others 60% | ART initiation | 86% | 13% | 4,387 | Mean:26.0 months | 135 | - |
| Estrella, M. M | 2015 | PAHO/ High | CKD (2s) | Low-Risk:42.7±8.3, High-risk:42.3±8.2 | 100.0% | African American | 73.9% | NR | NR | 333 | 7.6(IQR:6.7–8.0) years | 33 | - |
| Flandre P. | 2011 | EURO/ High | CKD (2s) | NR | 70.3% | NR | 89.3% | 34.0% | 27.8-43.1% | 7378 | 32319.9 | 349 | 10.8 |
| Ganesan A. | 2013 | PAHO/ High | CKD (2s) | 28.8 (IQR:24.5–34.1) | 92.4% | white 44.6%, black 44.1%, others 11.3% | time-updated covariate | NR | NR | 3360 | 23091 | 116 | 5.0 |
| Han, W.M. | 2020 | mixed | CKD (2) | age category | 68.3% | Asian | 100.0% | 60.90% | 40.20% | 6092 | 48875 | 391 | 8.0 |
| Hara, M. | 2017 | WPRO/ High | ①CKD (NR); ②≥30% decrease in eGFR | 46 ± 12 | 90.5% | Japanese94.1% | 90.2% | 48.7% | 35.9% | 661 | 6 years | 509 | - |
| Horberg, M. | 2010 | PAHO/ High | >50% decrease in eGFR (2s) | TDF: 43.0 (IQR:37.9-49.5); non-TDF:41.5 (35.0-49.0) | 85.3% | black 24.5%, others 75.5% | ART initiation | 58.5% | 44.0% | 1674 | TDF: 27.6 (IQR:19.7-39.6); non-TDF:32.6 (20.9, 43.7) | TDF:46; non-TDF:20 | - |
| Ibrahim, F | 2012 | EURO/ High | eGFR <30 for >3 months | eGFR category | 78.6% | black 24.7%, others 75.3% | 79.9% | NR | NR | 20045 | 124730 | 56 | 0.4 |
| Jones, R. | 2018 | EURO/ High | ①CKD (2s); ②eGFR<15 for > 3 months (2s) | east africa:36±9.0, southern Africa:36±9.0, west africa:36±9.8, caribbean:37±11.7 | 39.9% | black | 46.3% at baseline | NR | NR | 7764 | ①49599; ②50474 | ①231,②65 | ①4.7, ②1.3 |
| Joshi K. | 2018 | mixed | CKD (2s) | 35.6 (IQR:30.5-42.3) | 70.5% | Asian | 100.0% | 27.6% | 18.7% | 2547 | 10900.5 | 37 | 3.4 |
| Kabore N. F. | 2019 | AFRO/ Low | CKD (2s) | 37.3 (IQR:31.4–44.5) | 28.0% | black | ART initiation | 34.5% | 2.5% | 3124 | 14318 | 27 | 1.9 |
| Kalayjian R. C. | 2012 | PAHO/ High | ①CKD (2s);  ②eGFR <45 (2s);  ③eGFR <30 (2s) | 40 (IQR:37-42) | 81.3% | black 38.5%, others 61.5% | 100.0% | 60.0% | 40.0% | 3329 | ①10990; ②10303; ③10000 | ①106,②34, ③16 | ①9.6, ②3.3, ③1.6 |
| Kalemeera F. | 2021 | AFRO/ Upper middle | eGFR <50 (1) | NR | 39.6% | unclear but mainly black | 100% at baseline | 99.2% | 5.9% | 6744 | 16808 | 400 | 23.8 |
| Koh, H.M. | 2016 | WPRO/ Upper middle | >25% decrease in eGFR (1) | 44.4 (9.9) | 75.5% | Asian | TDF initiation | 100 | 25.30 | 440 | 558.3 | 67 | 120.0 |
| Lapadula G. | 2016 | EURO/ High | CKD (2s) | 44.1±8.4 | 69.0% | white 95.4%, black 2.5%, other 2.1% | TDF initiation | 100.0% | 75.6% | 6984 | 8006 | 191 | 23.9 |
| Laprise C. | 2013 | PAHO/ High | eGFR <90 for > 3 months (2) | median :39.3 | 96.2% | white 91.9%, black 2.8%, other 5.4% | 100.0% | 70.6% | NR | 1043 | 4285 | 271 | 63.2 |
| Lee, J. E. | 2019 | WPRO/ High | >25% decrease in eGFR | TDF:46.15 ± 13.16, ABC:44.75 ± 12.94 | 88.0% | Asian | 100.0% | 51.00% | 52.00% | 210 | 379.6 | 27 | 71.1 |
| Liu, F. | 2020 | WPRO/ Upper middle | ① eGFR <90 (1s);  ②CKD (1s) or annual decrease in eGFR >3 unit (1s) | no-TDF:27(IQR:24-32), TDF:30(IQR:25-36) | 95.4% | Asian | ART initiation | 36.3% | 5.1% | 823 | 10(IQR:2-20) months | ①178  ②451 | - |
| Low J. Z. | 2018 | WPRO/ Upper middle | CKD or >25% decrease in eGFR (1s) | 38.0 (IQR:32.0–46.0) | 89.5% | Asian | 49% naïve, all TDF initiation | 100.0% | 10.8% | 314 | 555.6 | 30 | 54.0 |
| Lucas G. M. | 2010 | AFRO/ Low | ①eGFR: 60–89;  ②CKD (NR) | 30 (IQR:25–36) | 35.4% | black | 0.0% | 0.0% | 0.0% | 1202 | 2583 | 84 | 32.5 |
| Lucas G. M. | 2013 | PAHO/ High | 1. CKD (2s); ②eGFR <15 for >90 days (2s) or last eGFR < 15; ③>25% decrease in eGFR to<60 for >90 days (2s) | 42 (IQR:35-49) | 85.6% | black 40.9%, others 59.1% | 45.9% | 10.2% | 15.0% | ①59236;②61646; ③61367 | ①332428; ②354358; ③343940 | ①6878②1098;③3945 | ①20.7;②3.04;③11.5 |
| Lucas G. M. | 2008 | PAHO/ High | CKD (3s) | 38 (IQR:32–43) | 68.0% | non-Hispanic African American 78.3%, white 21.7% | NR | NR | NR | 4259 | 18778 | 210 | 11.2 |
| Mapesi H. | 2018 | AFRO/ Lower middle | ①eGFR <90 (1s); ②eGFR: 30–59 (1s); ③eGFR <30 (1s) | 37.9 (IQR:31.6–45.6) | 33.3% | black | ART initiation | 90.1% | 0.3% | 921 | 1062 | ①117  ②21  ③18 | ①110.2,  ②19.8,  ③16.9 |
| Matłosz, B. | 2019 | EURO/ High | ①CKD (1s);  ②CKD(3s) | 30.2(IQR 25.9–35.5) | 68.9% | NR | 94% at baseline | ①70.8%,②30.0%^‡^ | 76.1% | 267 | 2593 | 52 | 20.1 |
| Medapalli R. K. | 2012 | PAHO/ High | eGFR <45 for >90 days (2s) | HIV only:51±10, HIV and DM:56±9 | 97.1% | black 46.4%, non-black 53.6% | 21% at baseline | 23.1% | 50.3% | 12422 | NR | 1136 | - |
| Mills, A.M. | 2020 | PAHO/ High | CKD (2) | age category | ①84.1%, ②  85.1%, ③84.1% | ①black 40.4%,others 59.6%,  ②black 39.3%,others 60.7%,  ③black 40.3%,others 59.7% | ①40.20%, ②38.2%,③40.2% | - | - | ①22748, ②19444, ③22727 | ①107545.5,②90547.9, ③109444.4 | ①1183, ②661, ③1182 | ①11.0, ②7.3, ③10.8 |
| Mocroft A. | 2020 | mixed | CKD (2s) or >25% decrease in eGFR (2s) | 43(IQR:37–51) | 74.0% | white 85.1%, others 14.9% | 88.5% ever used at baseline | NR | NR | 14754 | 115335 | 1130 | 9.8 |
| Monteagudo-Chu M. O. | 2012 | PAHO/ High | eGFR <90 (1) | TDF:52.6±7.5, ABC:50.2±7.8 | 99.6% | black 56.5%, others 43.5% | 80.4% at baseline, initiation | 48.3% | 35.7% | 230 | NR | 55 for TDF, 44 for ABC | - |
| Morlat P. | 2013 | EURO/ High | CKD (2) | age category | 74.4% | NR | 95.8% ever at baseline | 75.1% | 71.5% | 4350 | 21983 | 209 | 9.5 |
| Nishijima, T. | 2012 | WPRO/ High | >25% decrease in eGFR (2) | TDF:36 (IQR:31–44), ABC:37 (IQR:31–43) | 97.8% | Asian | ART initiation | 39.6% | 5.0% | 503 | 447.2 | TDF:44, ABC:41 | 190.1 |
| Nishijima, T. | 2014 | WPRO/ High | ①Absolute decrease in eGFR >10 unit (2); ②>25% decrease in eGFR (2); ③CKD (2) | 36 (IQR:31–43) | 97.0% | Asian | 100% at baseline | 53.3% | 89.0% | 792 | TDF:1347.5, ABC: 1379.3 | ①613  ②269  ③40 | ①224.8,  ②98.7,  ③14.7 |
| Nishijima, T. | 2011 | WPRO/ High | >25% decrease in eGFR (2) | 38(IQR:33–46) | 95.2% | Asian | 58% at baseline, TDF initiation | 100.0% | 74.1% | 495 | 923.8 | 97 | 10.5 |
| Nishijima, T. | 2016 | WPRO/High | ①Absolute decrease in eGFR >20 unit (1); ②Absolute decrease in eGFR >10 unit (2s); ③>25% decrease in eGFR (1); ④CKD (1) | 38(32-45) | 96.0% | Asian 96%, others 4% | 37%, TDF initiation | 100.0% | 81% | 655 | 3.32 (IQR:2.02–5.31) | ①528;②44; ③20; ④2 | - |
| Ojeh B. V. | 2018 | AFRO/ Lower middle | CKD (1) | 39±9.0 | 32.9% | black | ART initiation | 61.0% | NR | 5273 | 3217.4 TDF-exposed, 2043.5 TDF-unexposed | 148 TDF, 47 non-TDF | TDF:46.0, non-TDF:23.0 |
| Pongpirul W. | 2018 | SEARO/ Upper middle | CKD (2s) | mean:39.96 | 58.5% | Asian | 100.0% | NR | NR | 5430 | 22035 | 229 | 10.4 |
| Pujari, S. N | 2014 | SEARO/ Lower middle | CKD (1) | 38 (IQR:34-45) | 75.8% | Unclear/Asian | 56.6%, TDF initiation | 100.0% | 25.5%- | 708 | 2 years | UK:8,  India:11 | - |
| Quesada P. R. | 2015 | EURO/ High | ①CKD (2s);  ②eGFR <30 (2s) | 41.4 ± 8.9 | 70.1% | unclear but mainly white | 62.4%, TDF initiation | 100.0% | 48.6% | 451 | ①2226; ②2272.7 | ①65  ②5 | ①29.2;  ②2.2 |
| Rasch M. G. | 2012 | EURO/ High | CKD (2) | 38.7 (IQR:31.9-46.8) | 85.2% | unclear but mainly white | 100.0% | NR | NR | 2044 | 14744 | 164 | 11.1 |
| Rockwood N. | 2012 | EURO/ High | CKD (1) | 43.0 ±9.5 | 87.0% | white 71.9%, black 12.2%, others 15.9% | 86%, ART initiation | 39.5% | 43.0% | 2115 | 2680 | 386 | 144.0 |
| Rossi C. | 2017 | PAHO/ High | CKD (2s) | 40 (IQR:33-46) | 85.0% | white 45.5%, black 14.2%, unknown 40.3% | ART initiation | 54.0% | 26-44% | 2595 | 10903 | 150 | 13.8 |
| Scherzer R. | 2012 | PAHO/ High | ①CKD (2s);  ②Annual decline in eGFR >3 unit for >2 years (2s) | no-TDF:47(IQR:40-52), TDF:45(IQR:39-52) | 97.7% | white 41.4%, black 49.2%, others 9.4% | TDF initiation | 100.0% | NR | ①10161;②10841 | ①51589;②56416 | ①533; ②3078 | ①10.3; ②54.6 |
| Scherzer, R. | 2014 | PAHO/ High | CKD (1) | TDF:47 (40, 53), non-TDF:48 (42, 54) | 100.0% | black 53.3%, others 46.7% | ART initiation | 68% | - | 21590 | 193771 | 2059 | 10.6 |
| Sutton S. S. | 2020 | PAHO/ High | CKD or >25% decrease in eGFR (2s) | TDF:48.89±11.62, no-TDF:52.5±12.43 | 97.0% | white 47.8%, black 46.4%, Asian0.9%, other/unknown 4.9% | 100.0% | 79.7% | 27.1% | 5811 | TDF:24962.4, no-TDF:5079.0 | TDF:664, no-TDF:225 | TDF:26.6, no-TDF:44.3 |
| Suzuki S. | 2017 | WPRO/ High | ①CKD (2s); ②eGFR <45 (2s); ③eGFR <30 (2s) | 37 (IQR:31–44) | 94.0% | Asian | 99.7% | 52.1% | 83.0% | 1383 | ①7281.6; ②7382.6; ③7142.9 | ①150;②11; ③1 | ①20.6;②1.5; ③0.1 |
| Tan, Q. | 2019 | WPRO/ Upper middle | ①eGFR <90 or >25% decrease in eGFR (1); ②Absolute decrease in eGFR >10 unit (1) | 29 (IQR:25-34) | 97.0% | Asian | TDF initiation | 100.0% | NR | 258 | 168 weeks | ①3;  ②64 | - |
| Tordato, F. | 2011 | EURO/ High | >20% decrease in eGFR (2s) | 39 (IQR:33.5-44) | 70.0% | white | ART initiation | 40% (Person-years of use) | 39% (Person-years of use) | 644 | 1412 | 96 | 70.0 |
| Wong C. | 2017 | PAHO/ High | CKD (2) | age category | 83.3% | black 49.3%, others 50.7% | 76.1% | NR | NR | 52411 | 298435 | 1785 | 2.6 |
| Woolnough, E.L. | 2018 | mixed | CKD (2) | 46(39-53) | 90.9% | white 86.9%, black 4.7%, Asian 6.0%, others 2.3% | 92.60% | 63.20% | 83.90% | 748 | 4.7(2.2-6.2) | 37 | - |
| Zachor H. | 2016 | AFRO/ Upper middle | ①CKD (1s);  ②Annual decline in eGFR >3 unit (1s) | 41 (IQR:34–47) | 65.5% | unclear but mainly black | 100.0% | 100.0% | NR | 650 | median:54 (46.6–98.0) weeks | ①15; ②361 | - |

Note: eGFR, estimated glomerular filtration rate; CKD, chronic kidney disease; MDRD, modification of diet in renal disease; CKD-EPI, Chronic kidney disease Epidemiology collaboration; CG, Cockroft-Gault equation; AA, African-American; ART, antiretroviral therapy; TDF, tenofovir; PIs, protease inhibitors; ABC, abacavir. AFRO, African Region; PAHO, Region of the Americas; SEARO, South-East Asia Region; EURO, European Region; EMRO, Eastern Mediterranean Region; WPRO, Western Pacific Region. NR: not report.

^#^ Number in the parenthesis indicated the times of measurement for the outcome, where 1 was one measurement, 1s was at least one measurement, 2 was two times of measurement, 2s was at least two times of measurement, 3s was at least three times of measurement.

^※^Three samples were drawn using the same eligibility criteria but each using a different eGFR equation, specifically (1) MDRD,(2) C-G and (3) CKD-EPI.

*Strictly-defined CKD was 2 consecutive eGFR<60 mL/min/1.73 m^2^ for 3 months apart. While one measurement of eGFR< 60 mL/min/1.73 m^2^ was also classified as CKD in this study.

†Asian countries included Cambodia, China, India, Indonesia, Japan, Malaysia, Philippines, Singapore, South Korea, Thailand and Vietnam.

‡Contradictory data that would not be extracted.

**Supplementary Table 2. Newcastle-Ottawa Scale (NOS) for the assessment of the studies’ quality**

| **Author** | **Year** | **Total Score** | **Selection** | | | | **Comparability** | **Outcome** | | |
| --- | --- | --- | --- | --- | --- | --- | --- | --- | --- | --- |
|  |  |  | Representativeness of the exposed cohort | Selection of the non-exposed cohort | Ascertainment  of exposure | Demonstration that  outcome of interest  was not present at  start of study | Comparability of  cohorts on the  basis of the  design or analysis | Assessment  of outcome | Was follow-up  long enough  for outcomes  to occur | Adequacy of  follow-up of  cohorts |
| Alves, T. P. | 2010 | 7 | 1 | 1 | 1 | 0 | 2 | 1 | 1 | 0 |
| Ando, M. | 2011 | 6 | 0 | 1 | 1 | 1 | 1 | 1 | 0 | 1 |
| Bock P. | 2019 | 7 | 0 | 1 | 1 | 1 | 2 | 1 | 0 | 1 |
| Bouatou Y. | 2018 | 9 | 1 | 1 | 1 | 1 | 2 | 1 | 1 | 1 |
| Campbell, L. J. | 2009 | 7 | 1 | 1 | 1 | 0 | 2 | 1 | 1 | 0 |
| Chaisiri, K. | 2010 | 6 | 0 | 1 | 1 | 1 | 2 | 1 | 0 | 0 |
| Cheung J. | 2018 | 8 | 1 | 1 | 1 | 1 | 2 | 1 | 1 | 0 |
| De Waal, R. | 2017 | 7 | 1 | 1 | 1 | 0 | 2 | 1 | 1 | 0 |
| Dietrich, L. G. | 2020 | 8 | 1 | 1 | 1 | 1 | 2 | 1 | 1 | 0 |
| Ding Y. | 2019 | 9 | 1 | 1 | 1 | 1 | 2 | 1 | 1 | 1 |
| Domingo P. | 2019 | 7 | 1 | 1 | 1 | 1 | 2 | 1 | 0 | 0 |
| Eaton, E.F. | 2019 | 7 | 1 | 1 | 1 | 1 | 2 | 1 | 0 | 0 |
| Estrella, M. M | 2015 | 8 | 1 | 1 | 1 | 1 | 2 | 1 | 1 | 0 |
| Flandre P. | 2011 | 7 | 1 | 1 | 1 | 0 | 2 | 1 | 1 | 0 |
| Ganesan A. | 2013 | 9 | 1 | 1 | 1 | 1 | 2 | 1 | 1 | 1 |
| Jones, R.. | 2018 | 7 | 1 | 1 | 1 | 0 | 2 | 1 | 1 | 0 |
| Han, W.M. | 2020 | 8 | 1 | 1 | 1 | 1 | 2 | 1 | 1 | 0 |
| Hara, M. | 2017 | 7 | 0 | 1 | 1 | 1 | 2 | 1 | 1 | 0 |
| Horberg, M. | 2010 | 8 | 1 | 1 | 1 | 1 | 2 | 1 | 1 | 0 |
| Ibrahim, F | 2012 | 8 | 1 | 1 | 1 | 1 | 2 | 1 | 1 | 0 |
| Joshi K. | 2018 | 8 | 1 | 1 | 1 | 1 | 2 | 1 | 1 | 0 |
| Kabore N. F. | 2019 | 7 | 0 | 1 | 1 | 0 | 2 | 1 | 1 | 1 |
| Kalayjian R. C. | 2012 | 8 | 1 | 1 | 1 | 1 | 2 | 1 | 1 | 0 |
| Kalemeera F. | 2021 | 8 | 0 | 1 | 1 | 1 | 2 | 1 | 1 | 1 |
| Koh, H.M. | 2016 | 5 | 0 | 0 | 1 | 1 | 2 | 1 | 0 | 0 |
| Lapadula G. | 2016 | 9 | 1 | 1 | 1 | 1 | 2 | 1 | 1 | 1 |
| Laprise C. | 2013 | 9 | 1 | 1 | 1 | 1 | 2 | 1 | 1 | 1 |
| Lee, J. E | 2019 | 5 | 0 | 0 | 1 | 1 | 2 | 1 | 0 | 0 |
| Liu, F. | 2020 | 6 | 0 | 0 | 1 | 1 | 2 | 1 | 1 | 0 |
| Low J. Z. | 2018 | 7 | 0 | 0 | 1 | 1 | 2 | 1 | 1 | 1 |
| Lucas G. M. | 2010 | 7 | 1 | 1 | 1 | 0 | 2 | 1 | 1 | 0 |
| Lucas G. M. | 2013 | 8 | 1 | 1 | 1 | 1 | 2 | 1 | 1 | 0 |
| Lucas G. M. | 2008 | 7 | 1 | 1 | 1 | 1 | 1 | 1 | 1 | 0 |
| Mapesi H. | 2018 | 9 | 1 | 1 | 1 | 1 | 2 | 1 | 1 | 1 |
| Matłosz, B. | 2019 | 5 | 0 | 0 | 1 | 0 | 2 | 1 | 1 | 0 |
| Medapalli R. K. | 2012 | 7 | 1 | 1 | 1 | 1 | 2 | 1 | 0 | 0 |
| Mills, A.M., | 2020 | 7 | 1 | 1 | 1 | 1 | 2 | 1 | 0 | 0 |
| Mocroft A. | 2020 | 8 | 1 | 1 | 1 | 1 | 2 | 1 | 1 | 0 |
| Monteagudo-Chu M. O. | 2012 | 7 | 0 | 0 | 1 | 1 | 2 | 1 | 1 | 1 |
| Morlat P. | 2013 | 9 | 1 | 1 | 1 | 1 | 2 | 1 | 1 | 1 |
| Nishijima, T. | 2012 | 7 | 0 | 1 | 1 | 1 | 2 | 1 | 1 | 0 |
| Nishijima, T. | 2014 | 7 | 0 | 1 | 1 | 1 | 2 | 1 | 1 | 0 |
| Nishijima, T. | 2016 | 8 | 1 | 0 | 1 | 1 | 2 | 1 | 1 | 1 |
| Nishijima, T. | 2011 | 8 | 0 | 1 | 1 | 1 | 2 | 1 | 1 | 1 |
| Ojeh B. V. | 2018 | 7 | 0 | 0 | 1 | 1 | 2 | 1 | 1 | 1 |
| Pongpirul W. | 2018 | 8 | 1 | 1 | 1 | 1 | 2 | 1 | 1 | 0 |
| Pujari, S. N | 2014 | 5 | 0 | 0 | 1 | 1 | 2 | 1 | 0 | 0 |
| Quesada P. R. | 2015 | 5 | 0 | 0 | 1 | 1 | 2 | 1 | 0 | 0 |
| Rasch M. G. | 2012 | 8 | 1 | 1 | 1 | 1 | 2 | 1 | 1 | 0 |
| Rockwood N. | 2012 | 6 | 0 | 0 | 1 | 1 | 2 | 1 | 1 | 0 |
| Rossi C. | 2017 | 8 | 1 | 1 | 1 | 1 | 2 | 1 | 1 | 0 |
| Scherzer R. | 2012 | 6 | 0 | 0 | 1 | 1 | 2 | 1 | 1 | 0 |
| Scherzer R. | 2014 | 7 | 0 | 1 | 1 | 1 | 2 | 1 | 1 | 0 |
| Sutton S. S. | 2020 | 8 | 1 | 1 | 1 | 1 | 2 | 1 | 1 | 0 |
| Suzuki S. | 2017 | 6 | 0 | 0 | 1 | 1 | 2 | 1 | 1 | 0 |
| Tan, Q. | 2019 | 6 | 0 | 0 | 1 | 1 | 2 | 1 | 0 | 1 |
| Tordato, F. | 2011 | 8 | 1 | 1 | 1 | 1 | 2 | 1 | 1 | 0 |
| Wong C. | 2017 | 8 | 1 | 1 | 1 | 1 | 2 | 1 | 1 | 0 |
| Woolnough, E.L. | 2018 | 8 | 1 | 1 | 1 | 1 | 2 | 1 | 1 | 0 |
| Zachor H. | 2016 | 7 | 0 | 0 | 1 | 1 | 2 | 1 | 1 | 1 |

Note: criteria for NOS items: (1) representativeness of the samples: one point was assigned if the subjects represent the HIV-infected individuals in a certain community; (2) selection of the non-exposed cohort: one point was assigned if the non-exposed cohort was drawn from the same community as the exposed cohort; (3) ascertainment of the exposure: one point was assigned if exposure was assessed through secure record or structured interview/physical examination; (4) demonstration that outcome of interest was not present at start of study; (5) comparability: two points were assigned for adequate adjustment of recognized risk factors for renal impairment in addition to age and sex, specially the ART use; one point for adjustment of age and sex, and zero point for no adjustment; (6) assessment of the outcome: one point was assigned if kidney impairment was recorded on the basis of medical records or register data; (7) follow-up duration: one point was assigned if the follow-up period lasted more than 3 years; (8) adequacy of following up of cohorts: one point was assigned if individuals’ follow up rate >60%, or if description was provided of those lost.

**Supplementary Table 3 Measurement of outcomes**

| **Outcome** | **Description of outcome** | **Reference** |
| --- | --- | --- |
| CKD (n=40) | eGFR <60 ml/min/ 1.73 m^2^ | Bock P. (2019), De Waal R. (2017), Domingo P. (2019), Eaton E.F. (2019), Estrella M. M. (2015), Hara M. (2017), Lucas G. M. (2010), Mapesi H. (2018), Nishijima T. (2016), Ojeh B. V. (2018), Pujari S. N. (2014), Quesada P. R. (2015), Suzuki S. (2017), Zachor H. (2016) |
|  | first eGFR < 60 ml/min per 1.73m^2^. | Rockwood N. (2012), Scherzer R. (2014) |
|  | at least one eGFR<60 mL/min/1.73 m^2^ | Matłosz B. (2019) |
|  | eGFR <60 ml/min/1.73 m^2^ and confirmed on a second measurement within 6 months | Bouatou Y. (2018) |
|  | eGFR<60mL/min ≥3 months | Campbell L. J. (2009), Ganesan A. (2013) |
|  | confirmed eGFR <60 ml/min/1.73 m^2^ ≥3 months | Cheung J. (2018) |
|  | two consecutive eGFR <60 mL/min/1.73 m^2^ ≥ 3 months | Flandre P. (2011), Joshi K. (2018), Kabore N. F. (2019), Nishijima T. (2014), Rasch M. G. (2012), Rossi C. (2017), Scherzer R. (2012), Wong C. (2017) |
|  | two consecutive eGFR<60 mL/min measured 3 to 6 months apart. | Lapadula G. (2016) |
|  | at least two consecutive eGFR<60 mL/min/1.73 m^2^ for >90 days | Woolnough E.L. (2018), Mills A.M. (2020) |
|  | at least two consecutive eGFR ≤60 mL/min/1.73 m^2^ at least 3 months apart. | Han, W.M. (2020) |
|  | at least two eGFR<60 mL/min/1.73 m^2^ >3 months | Jones R. (2018), Lucas G. M. (2008), Morlat P. (2013), Pongpirul W. (2018) |
|  | at least two GFR or last available GFR<60 ml/min/1.73 m^2^ for >90 days | Lucas G. M. (2013) |
| eGFR < 30 mL/min/1.73m^2^  (n=6) | eGFR <30 mL/min/1.73 m^2^ for >3 months | Ibrahim F. (2012) |
|  | eGFR < 30 mL/min/1.73 m^2^ | De Waal R. (2017), Kalayjian R. C. (2012), Mapesi H. (2018), Quesada P. R. (2015), Suzuki S. (2017) |
| eGFR < 90 mL/min/1.73m^2^ (n=6) | 60–89 ml/min/1.73m^2^ | Ding Y. (2019) |
|  | eGFR < 90 mL/min/1.73 m^2^ | Liu F. (2020), Lucas G. M. (2010), Mapesi H. (2018), Monteagudo-Chu M. O. (2012) |
|  | two consecutive eGFR <90 mL/min/1.73m^2^ ≥3 months | Laprise C. (2013) |
| Decrease in eGFR >25% (n=7) | 25% decrease in eGFR from the baseline | Chaisiri K. (2010) |
|  | more than 25% decrease in eGFR from the baseline | Lee J. E. (2019), Nishijima T. (2011), Nishijima T. (2012), Nishijima T. (2014), Nishijima T. (2016), Koh H.M. (2016) |
| Combination definition  (n=4) | the development of eGFR< 60 mL/min/1.73 m^2^ on consecutive measures ≥3 months or 25% decrease in at least one eGFR measure from baseline | Low J. Z. (2018) |
|  | confirmed (two measurements>3m apart) eGFR≤ 60 among those who baseline eGFR >60 or confirmed 25% decline in eGFR for persons with baseline eGFR ≤60ml | Ding Y. (2019), Mocroft A. (2020), Sutton S. S. (2020) |
| eGFR < 45 mL/min/1.73m^2^  (n=3) | eGFR <45 mL/min/1.73 m^2^ | Kalayjian R. C. (2012), Suzuki S. (2017) |
|  | eGFR < 45 ml/min/1.73 m^2^ confirmed on 2 outpatient measurements of serum creatinine≥90 days apart. | Medapalli R. K. (2012) |
| Decrease in eGFR>10 mL/min/1.73m^2^ (n=3) | decrease in eGFR > 10 ml/min/1.73m^2^ from the baseline | Nishijima T. (2014), Nishijima T. (2016), Tan Q. (2019) |
| eGFR < 15 mL/min/1.73m^2^ (n=2) | at least two eGFR<15 mL/min/1.73m^2^ >3 months | Jones R. (2018), Lucas G. M. (2013) |
| Decrease in eGFR>3 mL/min/1.73m^2^ (n=2) | an annual decline in eGFR≥ 3 ml/min/1.73m^2^ for two consecutive years. | Scherzer R. (2012) |
|  | an annual decline in eGFR> 3 mL/min | Zachor H. (2016) |
| Decrease in eGFR>50% (n=2) | decrease in eGFR ≥50% from the baseline | Alves T. P. (2010), Horberg M. (2010) |
| Progressive CKD(n=2) | ≥25% GFR decline from baseline to eGFR< 60 for >90 days. | Ando M. (2011), Lucas G. M. (2013) |
| Other definitions of kidney function | the third of 3 consecutive years with an eGFR drop of ≥5 mL/min/1.73m^2^ in each compared to the previous year. | Dietrich L. G. (2020) |
|  | decrease in eGFR ≥30 % from baseline | Hara M. (2017) |
|  | progress to CKD or annual decline in eGFR > 3 mL/min/1.73 m^2^ | Liu F. (2020) |
|  | two or more eGFR or last eGFR<15 ml/min/1.73 m^2^ >90 days | Lucas G. M. (2013) |
|  | decrease in eGFR >25% or eGFR <90mL/min/1.73 m^2^ | Tan Q. (2019) |
|  | confirmed decrease in eGFR >20% | Tordato F. (2011) |
|  | decrement in eGFR > 20ml/min per 1.73m^2^ | Nishijima T. (2016) |
|  | eGFR <50 mL/min/1.73m^2^ | Kalemeera F. (2021) |

Note: CKD, chronic kidney disease; eGFR, estimated glomerular filtration rate.

**Supplementary Figure 1. Funnel plot for incidence rate of eGFR <60 mL/min/1.73m^2^ among PLWH**

**Supplementary Figure 2. Sensitivity analysis for incidence rate of eGFR <60 mL/min/1.73m^2^ among PLWH**

**Supplementary Table 4. Summary of demographic risk factors of eGFR <60 mL/min/1.73m^2^ among PLWH**

| **Risk factor** | **Number of studies** | **Reference** |
| --- | --- | --- |
| Sex (Female vs. male) | 21 | Bock P. (2019), Bouatou Y. (2018), Cheung J. (2018), Domingo P. (2019), Ganesan A. (2013), Jones R. (2018), Joshi K. (2018), Lucas G. M. (2008), Matłosz B. (2019),Morlat P. (2013), Mocroft, A. (2015), Ojeh B. V. (2018), Pongpirul W. (2018), Pujari S. N (2014), Quesada P. R. (2015), Rasch M. G. (2012), Rockwood N. (2012), Rossi C. (2017), Suzuki S. (2017), Woolnough E.L. (2018), Zachor H. (2016) |
| Age (year) |  |  |
| *Per 1 year increase* | 10 | Bouatou Y. (2018), Cheung J. (2018), Domingo P. (2019), Lapadula G. (2016), Ojeh B. V. (2018), Pongpirul W. (2018), Quesada P. R. (2015), Rasch M. G. (2012), Scherzer R. (2014), Suzuki S. (2017) |
| *Per 5-year increase* | 4 | Ganesan A. (2013), Joshi K. (2018), Matłosz B. (2019), Rossi C. (2017) |
| *Per 10-year increase* | 5 | Cheung J. (2018), De Waal R. (2017), Jones R. (2018), Pujari S. N (2014), Zachor H. (2016) |
| Race |  |  |
| Black vs. non-black | 2 | Bouatou Y. (2018), Kalayjian R. C. (2012) |
| Black vs. white | 4 | Ganesan A. (2013), Lucas G. M. (2010), Rockwood N. (2012), Rossi C. (2017) |
| Others vs. white | 2 | Ganesan A. (2013), Rockwood N. (2012) |
| HCV |  |  |
| HCV co-infection | 13 | Bouatou Y. (2018), Flandre P. (2011), Joshi K. (2018), Kalayjian R. C. (2012), Lucas G. M. (2010), Morlat P. (2013), Ojeh B. V. (2018), Quesada P. R. (2015), Rasch M. G. (2012), Rockwood N. (2012), Rossi C. (2017), Woolnough E.L. (2018) |
| Category: HCV seronegative, HCV viremic, HCV aviremic | 1 | Lucas G. M. (2008) |
| HBV (yes vs. no) | 10 | Bouatou Y. (2018), Flandre P. (2011), Ganesan A. (2013), Joshi K. (2018), Lucas G. M. (2010), Morlat P. (2013), Quesada P. R. (2015), Rockwood N. (2012), Woolnough E.L. (2018), Zachor H. (2016) |
| Hypertension |  |  |
| Yes vs. no | 13 | Bouatou Y. (2018), Domingo P. (2019), Flandre P. (2011), Ganesan A. (2013), Joshi K. (2018), Koh H.M. (2016), Lapadula G. (2016), Morlat P. (2013), Mocroft A. (2015), Nishijima T. (2014), Nishijima T. (2016), Pongpirul W. (2018), Quesada P. R. (2015), Rossi C. (2017), Scherzer R. (2014), Suzuki S. (2017), Woolnough E.L. (2018), Zachor H. (2016) |
| Baseline status (yes vs. no) | 2 | Kabore N. F. (2019), Rasch M. G. (2012) |
| Pharmacologically treated | 1 | Kalayjian R. C. (2012) |
| **Diabetes mellitus** |  |  |
| Yes vs. no | 9 | Bouatou Y. (2018), Domingo P. (2019), Flandre P. (2011), Koh H.M. (2016), Lapadula G. (2016), Morlat P. (2013), Mocroft A. (2015), Nishijima T. (2014), Pongpirul W. (2018), Quesada P. R. (2015), Rossi C. (2017), Scherzer R. (2014), Woolnough E.L. (2018) |
| Baseline status (yes vs. no) | 1 | Rasch M. G. (2012) |
| Time-updated (yes vs. no) | 1 | Ganesan A. (2013) |
| Pharmacologically treated | 1 | Kalayjian R. C. (2012) |
| History (yes vs. no) | 2 | Woolnough E.L. (2018), Zachor H. (2016) |
| **BMI (kg/m^2^)** |  |  |
| *Per 1 unit increase* | 3 | Domingo P. (2019), Morlat P. (2013), Pongpirul W. (2018) |
| *Category* |  |  |
| Cut-off of 18.5 | 1 | Mapesi H. (2018) |
| Cut-off of 18.5, 25 and 30 | 1 | Bouatou Y. (2018) |
| Cut-off of 30 | 1 | Domingo P. (2019) |
| Weight (kg) |  |  |
| *Per 1 unit increase* | 2 | Nishijima T. (2014), Suzuki S. (2017) |
| *Per 10-unit increase* | 1 | Zachor H. (2016) |
| *Category* |  |  |
| Cut-off of 55 and 65 | 1 | Joshi K. (2018) |
| Cut-off of 60 | 1 | De Waal, R. (2017) |
| Height (cm, per 10-unit increase) | 1 | Zachor H. (2016) |
| Elevated WHR^**^ | 1 | Bouatou Y. (2018) |
| CKD risk score (cut-point of 1% and 5%) | 1 | Boyd M. A. (2017) |
| COPD (yes vs. no) | 1 | Domingo P. (2019) |
| Dyslipidemia | 5 | Bouatou Y. (2018), Domingo P. (2019), Morlat P. (2013), Nishijima T. (2014), Quesada P. R. (2015), Scherzer R. (2014) |
| History of CVD events | 1 | Domingo P. (2019), Mocroft A. (2015) |
| History of renal failure (yes vs. no) | 1 | Zachor H. (2016) |
| Hypercholesterolemia | 1 | Pongpirul W. (2018) |
| Urinary β2 macroglobulin | 1 | Nishijima T. (2016) |
| Proteinuria | 2 | Scherzer R. (2014), Woolnough E.L. (2018) |
| Liver fibrosis (APRI ≥1.5) | 1 | Rossi C. (2017) |
| Ln HDL cholesterol | 1 | Boyd M. A. (2017) |
| Ln total cholesterol | 1 | Boyd M. A. (2017) |
| Study region/area/spot | 3 | Bock P. (2019), Jones R. (2018), Pujari S. N (2014) |
| Tuberculosis (yes vs. no) | 2 | Bock P. (2019), De Waal R. (2017) |
| Lipodystrophy |  |  |
| Yes vs. no | 1 | Bouatou Y. (2018) |
| Category (none, fat loss, fat accumulation and fat loss and accumulation) | 1 | Bouatou Y. (2018) |
| Smoker |  |  |
| Yes vs. no | 3 | Bouatou Y. (2018), Domingo P. (2019), Quesada P. R. (2015) |
| Current smoker (yes vs. no) | 1 | Boyd M. A. (2017) |
| Ex-smoker (yes vs. no) | 1 | Boyd M. A. (2017) |
| Use of nephrotoxic drugs |  |  |
| Yes vs. no | 3 | Nishijima T. (2014), Ojeh B. V. (2018), Suzuki S. (2017) |
| Current use (yes vs. no) | 1 | Joshi K. (2018) |
| Baseline eGFR, ml/min/1.73m^2^ |  |  |
| *Per 1 unit increase* | 5 | Bouatou Y. (2018), Cheung J. (2018), Koh H.M. (2016), Nishijima T. (2014), Ojeh B. V. (2018), Suzuki S. (2017) |
| *Per 5-unit increase* | 1 | Matłosz, B. (2019) |
| *Per 10-unit increase* | 4 | Cheung J. (2018), Kalayjian R. C. (2012), Rossi C. (2017), Zachor H. (2016) |
| *category* |  |  |
| Cut-point of 75 | 1 | De Waal, R. (2017) |
| Cut-point of 90 | 3 | Bock P. (2019), Joshi K. (2018), Koh H.M. (2016) |
| Cut-point of 75 and 90 | 1 | Campbell, L. J. (2009) |
| Cut-point of 90 and 120 | 1 | Woolnough E.L. (2018) |
| Cut-point of 70, 80 and 85 | 1 | Rockwood N. (2012) |
| Baseline creatine clearance (ml/min, cut-point of 60, 70, 80 and 90) | 1 | Morlat P. (2013) |
| Baseline serum creatine | 1 | Quesada P. R. (2015) |
| History of abnormal creatinine | 1 | Zachor H. (2016) |
| Occurrence of acute kidney injury | 1 | Flandre P. (2011) |
| Higher eGFR before TDF introduction | 1 | Lapadula G. (2016) |
| Higher eGFR drop between TDF introduction and study baseline | 1 | Lapadula G. (2016) |
| Longer TDF exposure prior to onset of mild renal | 1 | Lapadula G. (2016) |
| Follow-up time with CD4 count ≤200 cells/μL, stratified by the D:A:D CKD Risk Score | 1 | Ryom L. (2020) |
| Effect of age, sex, and race | 1 | Wong C. (2017) |
| Effect of age 50 and sex | 1 | Koh H.M. (2016) |
| Framingham Cardiovascular Risk | 1 | Domingo P. (2019) |
| 1 unit change | 1 | Domingo P. (2019) |
| Category (low, moderate and high) | 1 | Domingo P. (2019) |
| Cardiovascular disease (yes vs. no) | 1 | Quesada P. R. (2015) |
| Comorbid conditions (yes vs. no) | 1 | Ojeh B. V. (2018) |

Note: HCV, hepatitis C virus; BMI, body mass index; eGFR, estimated glomerular filtration rate; CKD, chronic kidney disease; COPD, chronic obstructive pulmonary disease; CVD, cardiovascular diseases; APRI, aspartate aminotransferase-to-platelet ratio index; HDL, high density lipoproteins; TDF, tenofovir.

*1 article categorized by 40-year-old

† Not pooled for different criteria applied for dysli*p*idemia in different studies.

‡1 article categorized by eGFR was excluded in pooled estimates of odds ratio.

** 0.85 for men and 0.9 for women.

**Supplementary Table 5. Summary of HIV-related risk factors of eGFR <60 mL/min/1.73m^2^ among PLWH**

| **Risk factor** | **Number of studies** | **Reference** |
| --- | --- | --- |
| AIDS |  |  |
| Yes vs. no | 5 | Domingo P. (2019), Flandre P. (2011), Lucas G. M. (2010), Morlat P. (2013), Quesada P. R. (2015) |
| Baseline status (yes vs. no) | 3 | Bouatou Y. (2018), Kalayjian R. C. (2012), Rasch M. G. (2012) |
| Time-varying (yes vs. no) | 1 | Jones, R. (2018) |
| WHO stage 3/4 vs. stage 1/2 | 1 | Mapesi H. (2018) |
| Current CDC category of stage 1, 2 and 3 | 1 | Joshi K. (2018) |
| HIV infection route |  |  |
| IDU vs. heterosexuals | 3 | Bouatou Y. (2018), Domingo P. (2019), Lapadula G. (2016) |
| MSM vs. heterosexuals | 4 | Bouatou Y. (2018), Cheung J. (2018), Domingo P. (2019), Joshi K. (2018), |
| Other vs. heterosexuals | 3 | Bouatou Y. (2018), Domingo P. (2019), Jones R. (2018) |
| IDU/other vs. heterosexuals | 1 | Joshi K. (2018) |
| IDU vs. MSM | 1 | Cheung J. (2018) |
| Other vs. MSM | 1 | Cheung J. (2018) |
| IDU vs. non-IDU | 2 | Lucas G. M. (2010), Morlat P. (2013) |
| HIV diagnosis |  |  |
| *Duration* |  |  |
| Per 1 year | 5 | Campbell L. J. (2009), Domingo P. (2019), Koh H.M. (2016), Pujari S. N (2014), Woolnough E.L. (2018) |
| *Category* |  |  |
| Cut-point of calendar year 1995 | 1 | Rasch M. G. (2012) |
| Cut-point of calendar year 1996 | 1 | Ganesan A. (2013) |
| Delay since HIV diagnosis: cut-point of 5, 10 and 15 years | 1 | Morlat P. (2013) |
| CD4 count, cells/μL |  |  |
| *At baseline* |  |  |
| Per 1 cell/μLincrement | 2 | Suzuki S. (2017), Koh H.M. (2016) |
| Per 10 cell/μLincrement | 1 | Cheung J. (2018) |
| Per 100 cell/mm3 increment | 1 | Cheung J. (2018) |
| Ln base CD4 count | 1 | Boyd M. A. (2017) |
| Cut-off of 200 | 4 | Pongpirul W. (2018), Rasch M. G. (2012), Zachor H. (2016), Scherzer R. (2014) |
| Cut-off of 50 and 100 | 1 | Koh H.M. (2016) |
| Cut-off of 50, 100 and 200 | 1 | De Waal, R. (2017) |
| Cut-off of 100 and 300 | 1 | Lucas G. M. (2008) |
| Cut-off of 200, 350 and 500 | 1 | Bock P. (2019) |
| Cut-off of 258, 383 and 550 | 1 | Rockwood N. (2012) |
| *At HIV diagnosis* |  |  |
| Cut-off of 200, 350 and 500 | 1 | Ganesan A. (2013) |
| *Current/ during follow-up* |  |  |
| Per 50 cell/μLincrement | 1 | Jones, R. (2018) |
| Per 100 cell/μL increment | 3 | Kalayjian R. C. (2012), Pujari S. N (2014), Rossi C. (2017) |
| Cut-off of 200 and 350 | 1 | Bouatou Y. (2018) |
| Cut off of 350 and 500 | 1 | Joshi K. (2018) |
| Cut-off of 200, 350 and 500 | 1 | Morlat P. (2013) |
| Other cut-offs (≥750 vs. <50) | 2 | Ryom L. (2020), Woolnough E.L. (2018) |
| *Unclear* |  |  |
| Per 1 cell/μL increment | 1 | Nishijima, T. (2014) |
| Per 100 cell/μL increment | 1 | Domingo P. (2019) |
| Nadir CD4 count, cells/μL |  |  |
| Per 1 cell/μL increment | 1 | Matłosz, B. (2019) |
| Per 10 cell/μL increment | 1 | Cheung J. (2018) |
| Per 50 cell/μL increment | 1 | Jones, R. (2018) |
| Per 100 cell/μL increment | 4 | Cheung J. (2018), Domingo P. (2019), Kalayjian R. C. (2012), Pujari S. N (2014), |
| Cut-off of 200 | 1 | Mocroft A. (2015) |
| Cut-off of 100 and 200 | 1 | Joshi K. (2018) |
| Cut-off of 200, 350 and 500 | 1 | Ganesan A. (2013) |
| Other cut-offs (≥500 vs. <50) | 2 | Ryom L. (2020), Woolnough E.L. (2018) |
| Viral load (copies/mL) |  |  |
| Cut-off of 50 | 3 | Domingo P. (2019), Morlat P. (2013), Woolnough E.L. (2018) |
| Cut-off of 50 and 500 | 1 | Rockwood N. (2012) |
| Cut-off of 1000 | 1 | Bouatou Y. (2018) |
| Per 1 log change | 3 | Domingo P. (2019), Pongpirul W. (2018), Rossi C. (2017) |
| Current detectable vs. undetectable | 1 | Joshi K. (2018) |
| Detectable on ART | 1 | Matłosz, B. (2019) |
| Last pre-ART VL, per 1 log increase | 1 | Kalayjian R. C. (2012) |
| Post pre-ART VL (time-varying), per 1 log increase | 1 | Kalayjian R. C. (2012) |
| Calendar year of ART start | 5 | Bock P. (2019), Cheung J. (2018), Lucas G. M. (2010), Matłosz B. (2019), Rossi C. (2017) |
| ART use |  |  |
| *Yes vs. no* | 3 | Domingo P. (2019), Quesada P. R. (2015), Rasch M. G. (2012) |
| *Cumulative use* |  |  |
| Per day increase | 1 | Suzuki S. (2017) |
| Per 1 year increase | 2 | Domingo P. (2019), Matłosz, B. (2019) |
| Cut-off of 1, 2 and 3 years | 1 | Bouatou Y. (2018) |
| *Years on treatment at baseline* |  |  |
| >3 months | 1 | Bock P. (2019) |
| Per 1 year | 2 | Campbell L. J. (2009), Cheung J. (2018) |
| Per 5 years | 1 | Campbell, L. J. (2009) |
| *Proportion of time on HAART by 20% increase* | 1 | Ganesan A. (2013) |
| Exposure to NRTI |  |  |
| Yes vs. no | 1 | Morlat P. (2013) |
| Exposure to NNRTI | 2 | Morlat P. (2013), Quesada P. R. (2015) |
| TDF use |  |  |
| *Yes vs. no* | 5 | Ojeh B. V. (2018), Rasch M. G. (2012), Suzuki S. (2017), Scherzer R. (2014), Woolnough E.L. (2018) |
| *Ever use (yes vs. no)* | 3 | Matłosz B. (2019), Morlat P. (2013), Scherzer R. (2012) |
| *Current use (yes vs. no)* | 2 | Jones R. (2018), Joshi K. (2018) |
| *Cumulative use* |  |  |
| Per 1 year | 5 | Quesada P. R. (2015), Rockwood N. (2012), Rossi C. (2017), Scherzer R. (2012), Woolnough E.L. (2018) |
| Cut-off of never, 0.5, 1 and 3 years | 1 | Scherzer R. (2012) |
| *At baseline* |  |  |
| Per 1 year | 1 | Cheung J. (2018) |
| Per 5 years | 1 | Cheung J. (2018) |
| *Previous exposure* | 2 | Flandre P. (2011), Rockwood N. (2012) |
| *TDF (vs. before HAART exposure)* | 1 | Rasch M. G. (2012) |
| PIs use |  |  |
| Yes vs. no | 3 | Domingo P. (2019), Koh H.M. (2016), Morlat P. (2013), Quesada P. R. (2015) |
| Ever use (yes vs. no) | 1 | Pujari, S. N (2014) |
| Current use (yes vs. no) | 3 | Jones R. (2018), Joshi K. (2018), Pujari S. N (2014) |
| Baseline use (PIs vs. NNRTI) | 1 | De Waal, R. (2017) |
| RTV-boosted (yes vs. no) | 2 | Nishijima T. (2014), Suzuki S. (2017) |
| RTV-boosted, per 1 year | 1 | Cheung J. (2018) |
| RTV-boosted, per 5 years | 1 | Cheung J. (2018) |
| AZT exposure |  |  |
| Yes vs. no | 1 | Woolnough E.L. (2018) |
| Ever use (yes vs. no) | 2 | Matłosz B. (2019), Rockwood N. (2012) |
| Past use (yes vs. no) | 1 | Flandre P. (2011) |
| Recent use (yes vs. no) | 1 | Flandre P. (2011) |
| Cumulative use, per 1 year | 1 | Scherzer R. (2012), Woolnough E.L. (2018) |
| IDV exposure |  |  |
| Yes vs. no | 1 | Woolnough E.L. (2018) |
| Baseline use, per 1 year | 1 | Cheung J. (2018) |
| Baseline use, per 5 years | 1 | Cheung J. (2018) |
| Previous exposure | 2 | Flandre P. (2011), Rockwood N. (2012) |
| Recent use (yes vs. no) | 1 | Flandre P. (2011) |
| Cumulative use, per 1 year | 3 | Campbell L. J. (2009), Scherzer R. (2012), Woolnough E.L. (2018) |
| ABC exposure |  |  |
| Ever use (yes vs. no) | 1 | Flandre P. (2011) |
| Recent use (yes vs. no) | 1 | Flandre P. (2011) |
| Cumulative use, per 1 year | 1 | Scherzer R. (2012) |
| 3TC exposure |  |  |
| Past use (yes vs. no) | 1 | Flandre P. (2011) |
| Recent use (yes vs. no) | 1 | Flandre P. (2011) |
| Cumulative use, per 1 year | 1 | Scherzer R. (2012) |
| LPV/r exposure |  |  |
| Yes vs. no | 1 | Woolnough E.L. (2018) |
| Ever use (yes vs. no) | 1 | Rockwood N. (2012) |
| Past use (yes vs. no) | 1 | Flandre P. (2011) |
| Recent use (yes vs. no) | 1 | Flandre P. (2011) |
| Cumulative use, per 1 year | 2 | Rossi C. (2017), Scherzer R. (2012), Woolnough E.L. (2018) |
| EFV exposure |  |  |
| Ever use (yes vs. no) | 1 | Rockwood N. (2012) |
| Past use (yes vs. no) | 1 | Flandre P. (2011) |
| Recent use (yes vs. no) | 1 | Flandre P. (2011) |
| Cumulative use, per 1 year | 1 | Scherzer R. (2012) |
| NVP exposure |  |  |
| Past use (yes vs. no) | 1 | Flandre P. (2011) |
| Recent use (yes vs. no) | 1 | Flandre P. (2011) |
| Cumulative use, per 1 year | 1 | Scherzer R. (2012) |
| DRV/r exposure (ever use, yes vs. no) | 1 | Rockwood N. (2012) |
| ddI exposure |  |  |
| Yes vs. no | 1 | Koh H.M. (2016) |
| Past use (yes vs. no) | 1 | Flandre P. (2011) |
| Recent use (yes vs. no) | 1 | Flandre P. (2011) |
| Cumulative use, per 1 year | 1 | Scherzer R. (2012) |
| d4T exposure |  |  |
| Past use (yes vs. no) | 1 | Flandre P. (2011) |
| Recent use (yes vs. no) | 1 | Flandre P. (2011) |
| Cumulative use, per 1 year | 1 | Scherzer R. (2012) |
| ATV exposure |  |  |
| Past use (yes vs. no) | 1 | Flandre P. (2011) |
| Recent use (yes vs. no) | 1 | Flandre P. (2011) |
| Cumulative use, per 1 year | 2 | Rossi C. (2017), Scherzer R. (2012) |
| DDC exposure |  |  |
| Past use (yes vs. no) | 1 | Flandre P. (2011) |
| Recent use (yes vs. no) | 1 | Flandre P. (2011) |
| FTC exposure |  |  |
| Past use (yes vs. no) | 1 | Flandre P. (2011) |
| Recent use (yes vs. no) | 1 | Flandre P. (2011) |
| SQV exposure |  |  |
| Past use (yes vs. no) | 1 | Flandre P. (2011) |
| Recent use (yes vs. no) | 1 | Flandre P. (2011) |
| NFV exposure |  |  |
| Past use (yes vs. no) | 1 | Flandre P. (2011) |
| Recent use (yes vs. no) | 1 | Flandre P. (2011) |
| FAPV exposure |  |  |
| Past use (yes vs. no) | 1 | Flandre P. (2011) |
| Recent use (yes vs. no) | 1 | Flandre P. (2011) |
| RTV exposure, per 1 year | 1 | Scherzer R. (2012) |
| Nelfinavir exposure, per 1 year | 1 | Scherzer R. (2012) |
| Fosamprenavir exposure, per 1 year | 1 | Scherzer R. (2012) |
| Saquinavir exposure, per 1 year | 1 | Scherzer R. (2012) |
| Amprenavir exposure, per 1 year | 1 | Scherzer R. (2012) |
| Zalcitabine exposure, per 1 year | 1 | Scherzer R. (2012) |
| Delavirdine exposure, per 1 year | 1 | Scherzer R. (2012) |
| Tipranavir exposure, per 1 year | 1 | Scherzer R. (2012) |
| ART combination regimen |  |  |
| *Category* |  |  |
| NRTI+ATZ/r or EFV or DRV/r or LPV/r or others | 1 | Rockwood N. (2012) |
| TDF or d4T with/without PI, ABC+ddI, AZT+PI vs. AZT | 1 | Kabore N. F. (2019) |
| TDF+AZT (vs. no-HAART exposure) | 1 | Rasch M. G. (2012) |
| TDF+PI (vs. no-HAART exposure) | 1 | Rasch M. G. (2012) |
| TDF and/or PIs regimen | 2 | Bouatou Y. (2018), Kalayjian R. C. (2012) |
| TDF+PI or NNRTI vs. NNRTI | 1 | Kalayjian R. C. (2012) |
| EFV/TDF/FTC, non-TDF/EFV, EVG/c/TDF/FTC, RPV/TDF/FTC, RTV-boosted PI+TDF/FTC | 1 | LaFleur, J (2018) |
| *Cumulative use* |  |  |
| TDF/PI-based regimen use (cut-point of 1, 2 and 3 years vs. other ART regimen) | 1 | Bouatou Y. (2018) |
| TDF with/without PIs use (cut-point of 6 and 12 months vs. never use) | 1 | Morlat P. (2013) |
| ART status and VL (ART-experienced or naïve combined with VL of cut-point of 50, 1000 and 10000) | 1 | Jones, R. (2018) |
| Time with VL<500 copies/mL | 1 | Flandre P. (2011) |
| Time with CD4>200 cells/uL | 1 | Flandre P. (2011) |

Note: CKD, chronic kidney disease; CDC, Center for Disease Control and Prevention; IDU, injection drug user; MSM, men having sex with men; ART, antiretroviral therapy; VL, viral load; HAART, highly active antiretroviral therapy; NRTI, nucleoside reverse transcriptase inhibitor; NNRTI, non-nucleoside reverse transcriptase inhibitors; TDF, tenofovir; PIs, protease inhibitors; RTV, ritonavir; AZT, zidovudine; IDV, indinavir; ABC, abacavir; 3TC, lamivudine; LPV/r, lopinavir/ritonavir; EFV, efavirenz; NVP, nevirapine; DRV/r, darunavir/ritonavir; ddI, didanosine; d4T, stavudine; ATV, atazanavir; DDC, Zalcitabine; FTC, emtricitabine; SQV, saquinavir; NFV, nelfinavir; FAPV, RPV, rilpivirine; EVG/c, elvitegravir/cobicistat

**Supplementary Table 6. Meta-regression result of eGFR<60 among PLWH**

| Variables | Coefficient, % (95% CI) | *p* value | Bonferroni-adjusted *p* |
| --- | --- | --- | --- |
| WHO region |  |  |  |
| American | -0.29(-1.34-0.75) | 0.564 | 1.000 |
| European | 0.22(-0.82-1.26) | 0.667 | 1.000 |
| South-East Asia | -0.26(-2.29-1.77) | 0.794 | 1.000 |
| Western Pacific | 0.26(-1.31-1.82) | 0.737 | 1.000 |
| Mixed | -0.58(-1.95-0.78) | 0.386 | 1.000 |
| Income level | |  |  |
| Lower middle | 0.78(0.63-3.85) | 0.008 | 0.040 |
| Upper middle | 0.77(0.55-3.74) | 0.011 | 0.055 |
| High | 0.58(0.32-2.73) | 0.015 | 0.075 |
| Mixed | 0.71(-0.53-2.42) | 0.197 | 0.985 |
| Race^†^ |  |  |  |
| Mainly black | -0.78(-1.79-0.22） | 0.12 | 0.600 |
| Mainly Asian | -0.97(-2.08-0.14) | 0.084 | 0.420 |
| Mixed | -0.95(-1.95-0.04) | 0.06 | 0.300 |
| Unknow | -0.71(-1.99-0.58) | 0.267 | 1.000 |
| Median or average age (yrs) | |  |  |
| ≥40 | 0.87(0.09-1.65) | 0.03 | 0.090 |
| Unknow | -0.20(-0.97-0.58) | 0.609 | 1.000 |
| Male ≥60 % | 0.08（-0.71-0.87） | 0.841 | 0.841 |
| ART status |  |  |  |
| ART-experienced≥70% at baseline | 0.30(-0.52-1.13) | 0.453 | 1.000 |
| ART/TDF initiation | 0.80(-0.04-1.64) | 0.061 | 0.244 |
| Unknow | -0.54(-2.36-1.28) | 0.547 | 1.000 |
| Sample size≥1000 | -0.56(-1.52-0.39) | 0.237 | 0.237 |
| eGFR calculation equation |  |  |  |
| MDRD | 0.56(-0.10-1.21) | 0.093 | 0.093 |
